# Development and Validation of a Prediction Model for Intracranial Aneurysm Rupture Risk

**DOI:** 10.1001/jamanetworkopen.2025.50772

**Published:** 2025-12-23

**Authors:** Soichiro Fujimura, Takeshi Yanagisawa, Genki Kudo, Toshiki Koshiba, Masaaki Suzuki, Hiroyuki Takao, Toshihiro Ishibashi, Hayato Ohwada, Shigeo Yamashiro, Maarten J. Kamphuis, Laura T. van der Kamp, Robert W. Regenhardt, Mervyn D. I. Vergouwen, Gabriel J. E. Rinkel, Aman B. Patel, Yuichi Murayama

**Affiliations:** 1 Department of Mechanical Engineering, Tokyo University of Science, Katsushika-ku, Tokyo, Japan; 2 Division of Innovation for Medical Information Technology, The Jikei University School of Medicine, Minato-ku, Tokyo, Japan; 3 Department of Neurosurgery, The Jikei University School of Medicine, Minato-ku, Tokyo, Japan; 4 Department of Industrial and Systems Engineering, Tokyo University of Science, Noda-shi, Chiba, Japan; 5 Faculty of Liberal Arts and Sciences, Chukyo University, Showa-ku, Nagoya-shi, Aichi, Japan; 6 Division of Neurosurgery, Department of Cerebrovascular Medicine and Surgery, Saiseikai Kumamoto Hospital, Minami-ku, Kumamoto, Japan; 7 Department of Neurology and Neurosurgery, University Medical Center Utrecht Brain Center, University Medical Center Utrecht, Utrecht University, Utrecht, the Netherlands; 8 Department of Radiology, University Medical Center Utrecht, Utrecht University, Utrecht, the Netherlands; 9 Department of Neurosurgery, Massachusetts General Hospital, Harvard Medical School, Boston

## Abstract

**Importance:**

Unruptured intracranial aneurysms (UIAs) affect 3.2% of the general population, and approximately 85% of subarachnoid hemorrhages result from their rupture. Despite their classification as low risk by prediction tools such as PHASES (population, hypertension, age, size of aneurysm, earlier subarachnoid hemorrhage from another aneurysm, and site of aneurysm) and the Unruptured Cerebral Aneurysm Study (UCAS), UIAs less than 10 mm are susceptible to rupture.

**Objective:**

To develop and externally validate a machine-learning model (MLM) predicting rupture risk of UIAs.

**Design, Setting, and Participants:**

This retrospective multicenter prognostic study analyzed UIAs from 4 institutions across 3 continents from January 2003 to November 2022. Each UIA was characterized by 29 clinical and 18 morphological variables. For model development, patients with UIAs were drawn from a large institutional cohort. Statistical analysis was performed from April 2024 to March 2025.

**Exposure:**

An MLM based on the Light Gradient Boosting Machine algorithm was trained, and performance was assessed for validation externally.

**Main Outcomes and Measures:**

The primary outcome was aneurysm rupture within 2 years after risk evaluation. Model performance was assessed using sensitivity, specificity, positive predictive value (PPV), negative predictive value (NPV), positive likelihood ratio (PLR), negative likelihood ratio (NLR), and the area under the receiver operating characteristic curve (AUROC) with 95% CIs.

**Results:**

Drawing from 11 579 UIAs from multiple institutions of 8846 patients, there were 2750 patients with 3312 UIAs in the development cohort (median [IQR] age, 65.3 [54.9-73.6] years; 1856 females [67.5%]) and 1153 patients with 1501 UIAs in the external cohort (median [IQR] age, 63.6 [53.9-70.9] years; 828 females [71.8%]), for whom the MLM demonstrated a robust performance in risk estimation. In the development cohort, 71 UIAs (2.1%) ruptured during 8.5 years’ median follow-up (IQR, 5.1-11.6 years), and in the external cohort, 48 UIAs (3.2%) ruptured during a median (IQR) follow-up of 5.4 (3.7-8.7) years. With a threshold of 0.52 for the MLM, performance in the development cohort for sensitivity was 0.78 (95% CI, 0.67-0.86); specificity, 0.82 (95% CI, 0.80-0.83); PPV, 0.09 (95% CI, 0.07-0.11); NPV, 0.99 (95% CI, 0.99-1.00); PLR, 4.23 (95% CI, 3.66-4.89); NLR, 0.28 (95% CI, 0.18-0.42); and AUROC, 0.88 (95% CI, 0.85-0.92) and in the external cohort, sensitivity was 0.90 (95% CI, 0.78-0.95); specificity, 0.70 (95% CI, 0.67-0.72); PPV, 0.09 (95% CI, 0.07-0.12); NPV, 1.00 (95% CI, 0.99-1.00); PLR, 2.94 (95% CI, 2.60-3.33); NLR, 0.15 (95% CI, 0.07-0.34); and AUROC, 0.90 (95% CI, 0.85-0.94). The MLM performed consistently in UIAs less than 10 mm (AUROC, 0.88 [95% CI, 0.82-0.94]), suggesting potential complementary value to PHASES and the UCAS.

**Conclusions and Relevance:**

In this prognostic study, the MLM consistently identified features of UIAs as significantly associated with rupture across different cohorts. These results support the MLM’s potential to assist physicians and patients in UIA treatment decisions.

## Key Points

**Question:**

Can a machine-learning model (MLM) predict the rupture risk of unruptured intracranial aneurysms (UIAs) using prerupture data?

**Findings:**

In this prognostic study of 11 579 UIAs from a cohort of 8846 patients, an MLM trained on prerupture clinical and morphological data demonstrated robust performance in both internal and external validation, including on aneurysms smaller than 10 mm.

**Meaning:**

The findings of this study suggest that an MLM may improve risk stratification and inform treatment decision-making for patients with UIAs, providing additional guidance even for smaller aneurysms traditionally considered low risk.

## Teaser

This prognostic study develops and validates a machine-learning model that predicts rupture risk of unruptured intracranial aneurysms.

## Introduction

Intracranial aneurysms (IAs) are outpouchings arising from localized weakness in the cerebral arterial wall. The overall prevalence of unruptured IAs (UIAs) is approximately 3.2%.^1^ Approximately 85% of subarachnoid hemorrhages (SAHs) result from IA rupture.^2,3^ An aneurysmal SAH has a 32% case-fatality rate after hospitalization,^4^ and 12% of patients die before reaching medical care.^5^ Approximately 50% of the survivors experience permanent neurological or cognitive deficits.^4,6^ The annual incidence of IA rupture is between 0.7% and 1.9%^7^ and is associated with factors such as size, location, morphology,^2,8,9,10^ environment, social habits, sex, family history, and race and ethnicity.^11,12,13,14,15,16,17^ These factors guide the risk-benefit assessment of preventive aneurysm treatment.

Several prediction tools, including PHASES (population, hypertension, age, size of aneurysm, earlier SAH from another aneurysm, and site of aneurysm)^18^ and the Unruptured Cerebral Aneurysm Study (UCAS),^19^ can be used to assess rupture risk. However, although UIAs less than 10 mm are classified by these tools as low risk, rupture has been observed in such aneurysms.^9,10,20,21,22,23^ More objective criteria are needed to identify which aneurysms warrant surgical or interventional treatment. Therefore, this study aimed to develop and validate a machine-learning model (MLM) to predict which UIAs, including those less than 10 mm, may rupture during follow-up.

## Methods

### Study Design

This multicenter prognostic study was conducted at 4 institutions in Japan, the US, and the Netherlands to establish a UIA database, including IAs ruptured during follow-up. The study was approved by the ethical committee of The Jikei University School of Medicine and was conducted in accordance with the Declaration of Helsinki^44^; the study protocol was approved but not publicly available. Written informed consent was obtained from participants when feasible, and an opt-out procedure, approved by the ethics committee, was applied otherwise. In all participating institutions, radiological monitoring was performed when the risk of preventive intervention was considered higher than that of natural rupture. For each aneurysm, the risk evaluation date was defined as the final imaging date within 2 years before the occurrence of a SAH (ruptured aneurysms) or the last imaging date 2 or more years before the end of the observation period (unruptured aneurysms). Data obtained up to this date were used to train an MLM to predict rupture within 2 years. The MLM was developed using the largest dataset from institution A (Japan) and subsequently applied to data from institution B (Japan), institution C (US), and institution D (Netherlands) to evaluate performance. Furthermore, we focused on UIAs less than 10 mm, which are traditionally considered low risk. The study followed the Transparent Reporting of a Multivariable Prediction Model for Individual Prognosis or Diagnosis (TRIPOD) reporting guideline.

### Data Collection

Data regarding patients with UIAs were retrospectively collected from outpatient aneurysm clinics at 4 centers between January 2003 and November 2022. The inclusion criteria were being aged 18 years or older, having a saccular aneurysm, and undergoing follow-up with magnetic resonance angiography (MRA), computed tomography angiography (CTA), or calibrated digital subtraction angiography (DSA). No competing risk framework was adopted, and patients whose primary outcome (ruptured or unruptured status) could be ascertained were included in this study. Data were collected, including age at the risk evaluation date, sex, ethnicity, aneurysm size, location, multiplicity, UIA count, weekly alcohol consumption, smoking status, and past medical history. Ethnicity was classified by the investigators based on medical record information as Finnish, Japanese, or other (including all patients not classified as Finnish or Japanese ethnicity), reflecting prior evidence from the PHASES score that identified an association with higher rupture risk in Finnish and Japanese populations.^18^ Ethnicity was assessed to examine potential differences in rupture risk across populations. The aneurysm location was categorized as internal carotid artery, middle cerebral artery, anterior cerebral artery, basilar artery, or vertebral artery. The PHASES and the UCAS scores of each aneurysm were calculated. For PHASES, scores range from 0 to 22, with higher scores indicating greater predicted rupture risk; for the UCAS, scores range from 0 to 15, with higher scores indicating greater predicted rupture risk. All eligible cases during the study period were included without a priori sample-size calculation. Details of exclusion criteria and additional patient characteristics are provided in eMethods 1 in Supplement 1.

### Morphological Aneurysm Characteristics

Aneurysms were measured by local investigators with expertise in UIAs according to the published methods.^24^ Three-dimensional CTA or DSA was primarily used at institutions A, B, and C, whereas 3-tesla MRA–time of flight was used at institution D. The following parameters were collected from all scans during the follow-up period, including the baseline and last scan: aneurysm length, width, neck diameter, and presence of irregularity. The aspect ratio and aneurysm saccularity (the ratio of IA size to the neck) were calculated: saccularity = (length × width)/(neck diameter).

### Time-Dependent Morphological Parameters

Time-dependent morphological parameters, including rate-length, rate-width, rate-neck, lr-length-a, lr-length-b, lr-length-R, lr-width-a, lr-width-b, lr-width-R, lr-neck-a, lr-neck-b, and lr-neck-R, were calculated for patients with 2 or more scans during follow-up. Rate parameters represented daily changes in length, width, and neck diameter: rate − parameter = (parameter value at last assessment − parameter value at initial assessment)/(days between initial and last assessment).

For lr parameters, *y* = *ax + b* was fit to each dimension (length, width, or neck) over time, extracting parameters *a*, denoted by the suffix a; *b*, denoted by b; and *R^2^*, denoted by R.

### Development of MLM and External Validation Design

The data were preprocessed to ensure consistency. Clinical variables lacking documentation were treated as missing, and time-dependent morphological parameters were marked as missing if only 1 measurement existed, allowing the model to accommodate aneurysms lacking longitudinal imaging. Categorical variables were encoded using label encoding, whereas continuous variables were standardized. A binary classification model (ruptured vs unruptured) was constructed using the Light Gradient Boosting Machine, with hyperparameter tuning performed using bayesian optimization (Optuna, version 4.2.1 [Preferred Networks, Inc]) and the F1 score as the evaluation metric.^25^ Training was performed using data from the largest aneurysm cohort (institution A). Because of the imbalance (ruptured vs unruptured), undersampling was performed using k-means clustering to preserve the distribution. The threshold derived from the MLM rupture risk indicator was applied to external data from institutions B, C, and D. Further methodologic details are provided in eMethods 2 in Supplement 1.

### Statistical Analysis

Clinical and morphological differences between ruptured and unruptured aneurysms were compared using Fisher exact or Mann-Whitney *U* tests. Continuous variables were presented as median (IQR), whereas categorical variables were presented as number (percentage). Two-sided *P* < .05 was considered statistically significant. The MLM performance was evaluated using sensitivity, specificity, positive predictive value (PPV), negative predictive value (NPV), positive likelihood ratio (PLR), negative likelihood ratio (NLR), and area under the receiver operating characteristic curve (AUROC). Furthermore, the AUROC of the MLM was compared with those of PHASES and the UCAS using the DeLong test. Calibration was evaluated using calibration-in-the-large, the calibration slope, the Brier score, and calibration curves. Logistic recalibration was performed in the development cohort to obtain a fixed calibrator, which was applied unchanged to external cohorts. Data were analyzed using SAS, version 9.4 (SAS Institute Inc) from April 2024 to March 2025.

## Results

**Figure 1.**
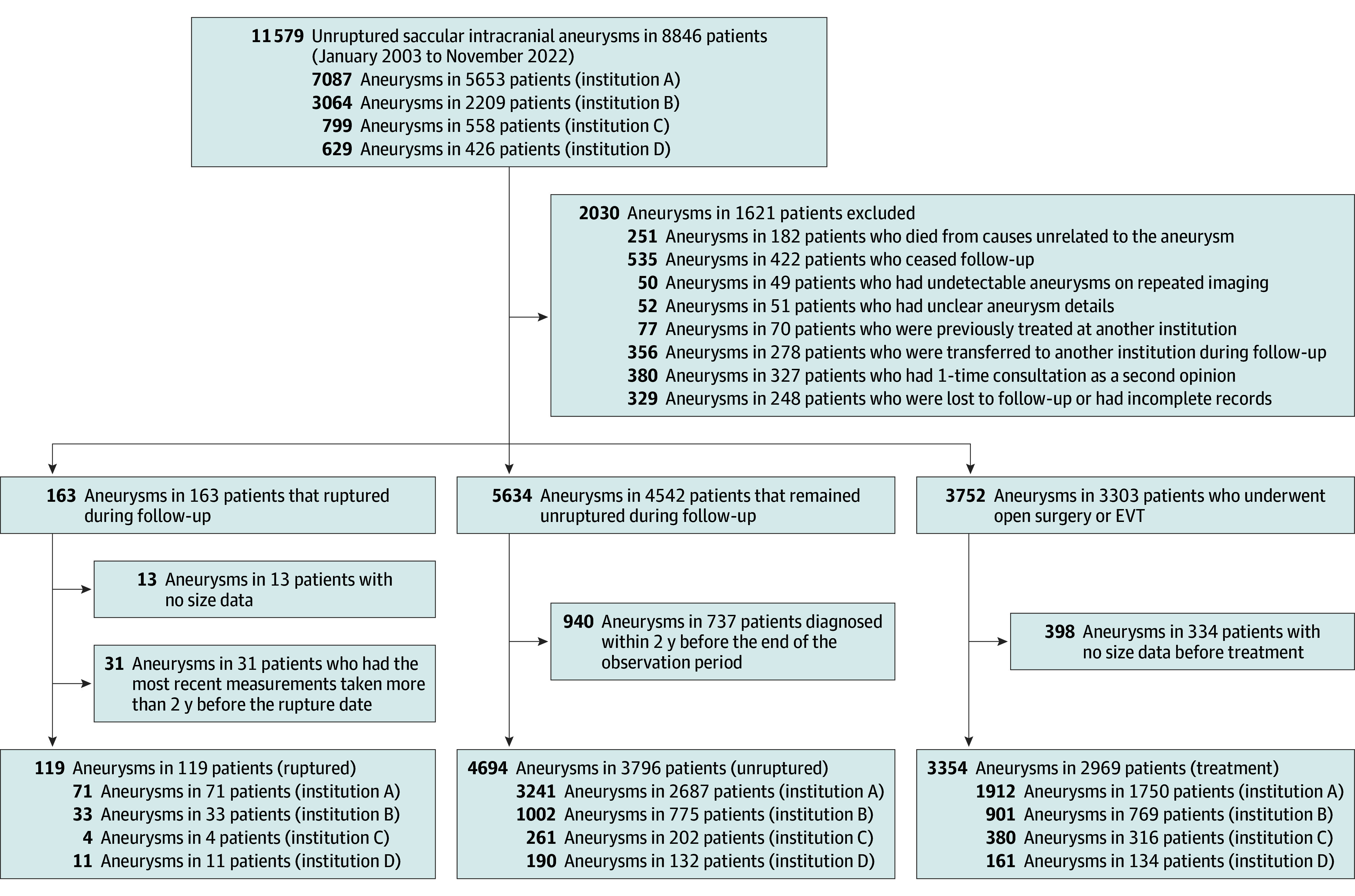
Flowchart Describing the Patient Selection Unruptured intracranial aneurysms registered during outpatient visits between January 2003 and November 2022 were included. The machine-learning models were developed and evaluated using aneurysms that met the inclusion criteria. EVT indicates endovascular treatment.

A total of 8846 patients with 11 579 UIAs from 4 institutions were identified (median [IQR] age, 64.8 [55.2-72.4] years; 6316 females [71.4%] and 2530 males [28.6%]) (Figure 1). Among them, 163 UIAs ruptured; 4813 UIAs in 3915 patients were eligible for analysis. Of the 4813 UIAs, 119 (2.5%) were ruptured aneurysms in 119 patients, and 4694 (97.5%) were unruptured aneurysms in 3796 patients during a follow-up of 37 865 aneurysm-years (annualized rupture rate, 0.31% per year). The baseline characteristics of the patients and aneurysms are shown in eTable 1 in Supplement 1. The median (IQR) length of the ruptured aneurysms was 5.8 (3.6-8.1) mm; 36 (30.3%) were located in the internal carotid artery, 20 (16.8%) in the middle cerebral artery, 35 (29.4%) in the anterior cerebral artery, 24 (20.2%) in the basilar artery, and 4 (3.4%) in the vertebral artery. Conversely, the median (IQR) length of the unruptured aneurysms was 2.7 (2.0-3.6) mm; 2588 (55.1%) were located in the internal carotid artery, 1083 (23.1%) in the middle cerebral artery, 667 (14.2%) in the anterior cerebral artery, 270 (5.8%) in the basilar artery, and 86 (1.8%) in the vertebral artery. No Finnish patients were included in the study population; therefore, the final ethnicity categories analyzed were Japanese (4320 [89.8%]) and other (493 [10.2%]). Additional details are provided in eResults 1 in Supplement 1.

### Development Cohort and the MLM

A total of 3312 UIAs in 2750 patients in the development cohort (institution A) were analyzed (median [IQR] age, 65.3 [54.9-73.6] years; 1856 females [67.5%] and 894 males [32.5%]). Of the 3312 UIAs, 71 (2.1%) ruptured over a cumulative 28 289 aneurysm-years of follow-up (0.25% per year), with a median (IQR) follow-up of 8.5 (5.1-11.6) years (eFigure 1 in Supplement 1). The baseline characteristics of the development cohort are shown in Table 1. Of the 3312 UIAs, 2540 had 2 or more imaging measurements, allowing for calculating time-dependent morphological parameters. Conversely, 772 UIAs had a single imaging measurement available. Nevertheless, all UIAs were included in the dataset for model training.

**Table 1.** Patient and Aneurysm Characteristics in the Development Cohort

Parameter	No. (%) or median (IQR)				*P* value^a^
	Patients, total (N = 2750)	Aneurysms			
		Total (N = 3312)	Ruptured (n = 71)	Unruptured (n = 3241)	
**Patient characteristics**					
Age, y	65.3 (54.9 to 73.6)	65.9 (55.4 to 73.9)	65.4 (52.7 to 73.5)	65.9 (55.5 to 73.9)	.52
Sex					
Female	1856 (67.5)	2277 (68.8)	53 (74.6)	2224 (68.6)	.28
Male	894 (32.5)	1035 (31.2)	18 (25.4)	1017 (31.4)	
Multianeurysms	817 (29.7)	1379 (41.6)	30 (42.3)	1349 (41.6)	.92
No. of aneurysms	1 (1 to 2)	1 (1 to 2)	1 (1 to 2)	1 (1 to 2)	.92
Hypertension	1204 (43.8)	1481 (44.7)	32 (45.1)	1449 (44.7)	.95
Alcohol consumption/wk	403 (14.7)	470 (14.2)	11 (15.5)	459 (14.2)	.76
Smoking	372 (13.5)	449 (13.6)	8 (11.3)	441 (13.6)	.57
No. of cigarettes/d	0 (0 to 10.0)	0 (0 to 10.0)	0	0 (0 to 10.0)	.29
y of Smoking	0	0	0	0	.41
Hyperlipidemia	480 (17.5)	577 (17.4)	8 (11.3)	569 (17.6)	.17
Diabetes	178 (6.5)	219 (6.6)	2 (2.8)	217 (6.7)	.19
SAH	70 (2.5)	82 (2.5)	9 (12.7)	73 (2.3)	<.001
Polycystic kidney disease	40 (1.5)	46 (1.4)	2 (2.8)	44 (1.4)	.30
Cerebral hemorrhage	5 (0.2)	6 (0.2)	1 (1.4)	5 (0.2)	.01
Cerebral infarction	4 (0.1)	5 (0.2)	0 (0)	5 (0.2)	.74
Family history of a SAH	338 (12.3)	416 (12.6)	10 (14.1)	406 (12.5)	.70
Family history of an aneurysm	63 (2.3)	77 (2.3)	2 (2.8)	75 (2.3)	.78
Family history of a polycystic kidney	12 (0.4)	13 (0.4)	0	13 (0.4)	.59
mRS (first visit)	0	0	0	0	.97
Ethnicity^b^					
Japanese	2726 (99.1)	3284 (99.2)	70 (98.6)	3214 (99.2)	.60
Other^c^	24 (0.9)	28 (0.8)	1 (1.4)	27 (0.8)	
**Aneurysm characteristics**					
Location					
ICA (all sites)	NA	1863 (56.3)	25 (35.2)	1838 (56.7)	<.001
ICA-Pcom	NA	603 (18.2)	18 (25.4)	585 (18.0)	.12
MCA (all sites)	NA	748 (22.6)	16 (22.5)	732 (22.6)	.99
ACA (all sites)	NA	469 (14.2)	17 (23.9)	452 (13.9)	.02
ACA-Acom	NA	310 (9.4)	10 (14.1)	300 (9.3)	.17
BA (all sites)	NA	191 (5.8)	11 (15.5)	180 (5.6)	<.001
BA-tip	NA	80 (2.4)	7 (9.9)	73 (2.3)	<.001
VA	NA	41 (1.2)	2 (2.8)	39 (1.2)	.22
Morphological parameters					
Presence of irregularity	NA	159 (4.8)	24 (33.8)	135 (4.2)	<.001
Length, mm	NA	2.8 (2.0 to 3.7)	5.2 (3.3 to 7.3)	2.8 (2.0 to 3.7)	<.001
Width, mm	NA	3.4 (2.7 to 4.4)	4.4 (3.6 to 6.9)	3.4 (2.7 to 4.4)	<.001
Neck diameter, mm	NA	3.3 (2.7 to 4.0)	3.8 (3.0 to 5.3)	3.3 (2.7 to 4.0)	.001
Aspect ratio​	NA	0.97 (0.80 to 1.15)	1.04 (0.83 to 1.34)	0.97 (0.81 to 1.14)	.06
Saccularity, mm	NA	3.4 (2.5 to 4.8)	5.9 (3.7 to 9.2)	3.3 (2.5 to 4.7)	<.001
Time-dependent morphological parameters					
Rate-length, mm/d	NA	2.1 × 10^−5^ (−1.1 × 10^−4^ to 2.2 × 10^−4^)	8.6 × 10^−4^ (5.5 × 10^−5^ to 3.8 × 10^−3^)	2.0 × 10^−5^ (−1.1 × 10^−4^ to 2.1 × 10^−4^)	<.001
Rate-width, mm/d	NA	4.8 × 10^−5^ (−1.7 × 10^−4^ to 3.1 × 10^−4^)	1.8 × 10^−4^ (−2.0 × 10^−4^ to 1.2 × 10^−3^)	4.7 × 10^−5^ (−1.7 × 10^−4^ to 3.0 × 10^−4^)	.15
Rate-neck, mm/d	NA	2.7 × 10^−5^ (−1.5 × 10^−4^ to 2.3 × 10^−4^)	3.8 × 10^−5^ (−7.1 × 10^−5^ to 9.5 × 10^−4^)	2.6 × 10^−5^ (−1.5 × 10^−4^ to 2.2 × 10^−4^)	.12
lr-length-a	NA	4.6 × 10^−2^ (3.3 × 10^−2^ to 6.1 × 10^−2^)	7.3 × 10^−2^ (5.5 × 10^−2^ to 9.4 × 10^−2^)	4.6 × 10^−2^ (3.3 × 10^−2^ to 6.1 × 10^−2^)	<.001
lr-length-b	NA	5.0 × 10^−4^ (−7.0 × 10^−4^ to 3.3 × 10^−3^)	−1.3 × 10^−3^ (−3.2 × 10^−3^ to 0)	5.0 × 10^−4^ (−6.0 × 10^−4^ to 3.3 × 10^−3^)	<.001
lr-length-R	NA	0.99 (0.98 to 1.0)	0.99 (0.96 to 1.00)	0.99 (0.98 to 1.00)	.16
lr-width-a	NA	5.4 × 10^−2^ (4.3 × 10^−2^ to 7.1 × 10^−2^)	7.8 × 10^−2^ (6.1 × 10^−2^ to 1.0 × 10^−1^)	5.4 × 10^−2^ (4.3 × 10^−2^ to 7.0 × 10^−2^)	<.001
lr-width-b	NA	4.0 × 10^−4^ (−1.1 × 10^−3^ to 3.5 × 10^−3^)	0 (−1.2 × 10^−3^ to −1.5 × 10^−3^)	4.0 × 10^−4^ (−1.1 × 10^−3^ to 3.5 × 10^−3^)	.17
lr-width-R	NA	0.99 (0.98 to 1.00)	0.99 (0.96 to 1.00)	0.99 (0.98 to 1.00)	.92
lr-neck-a	NA	5.4 × 10^−2^ (4.4 × 10^−2^ to 6.6 × 10^−2^)	5.8 × 10^−2^ (4.7 × 10^−2^ to 8.5 × 10^−2^)	5.4 × 10^−2^ (4.4 × 10^−2^ to 6.6 × 10^−2^)	.07
lr-neck-b	NA	8.0 × 10^−4^ (−3.0 × 10^−4^ to 4.2 × 10^−3^)	0 (−1.2 × 10^−3^ to 4.8 × 10^−4^)	8.0 × 10^−4^ (−3.0 × 10^−4^ to 4.3 × 10^−3^)	<.001
lr-neck-R	NA	0.99 (0.98 to 1.00)	1.00 (0.99 to 1.00)	0.99 (0.98 to 1.00)	.11

Abbreviations: a, *a* parameter; ACA, anterior cerebral artery; Acom, anterior communicating artery; b, *b* parameter; BA, basilar artery; ICA, internal carotid artery; lr, *y* = *ax + b*; MCA, middle cerebral artery; mRS, modified Rankin scale; NA, not applicable; Pcom, posterior communicating artery; R, *R^2^* parameter; SAH, subarachnoid hemorrhage; VA, vertebral artery.

^a^ Clinical and morphological differences between ruptured and unruptured aneurysms were compared using the Fisher exact test for categorical variables and the Mann-Whitney test for continuous variables.

^b^ Categories were investigator-defined as Finnish, Japanese, or other based on prior evidence from the PHASES (population, hypertension, age, size of aneurysm, earlier SAH from another aneurysm, and site of aneurysm) score, but no Finnish patients were included.

^c^ Includes all patients not classified as Finnish or Japanese.

**Figure 2.**
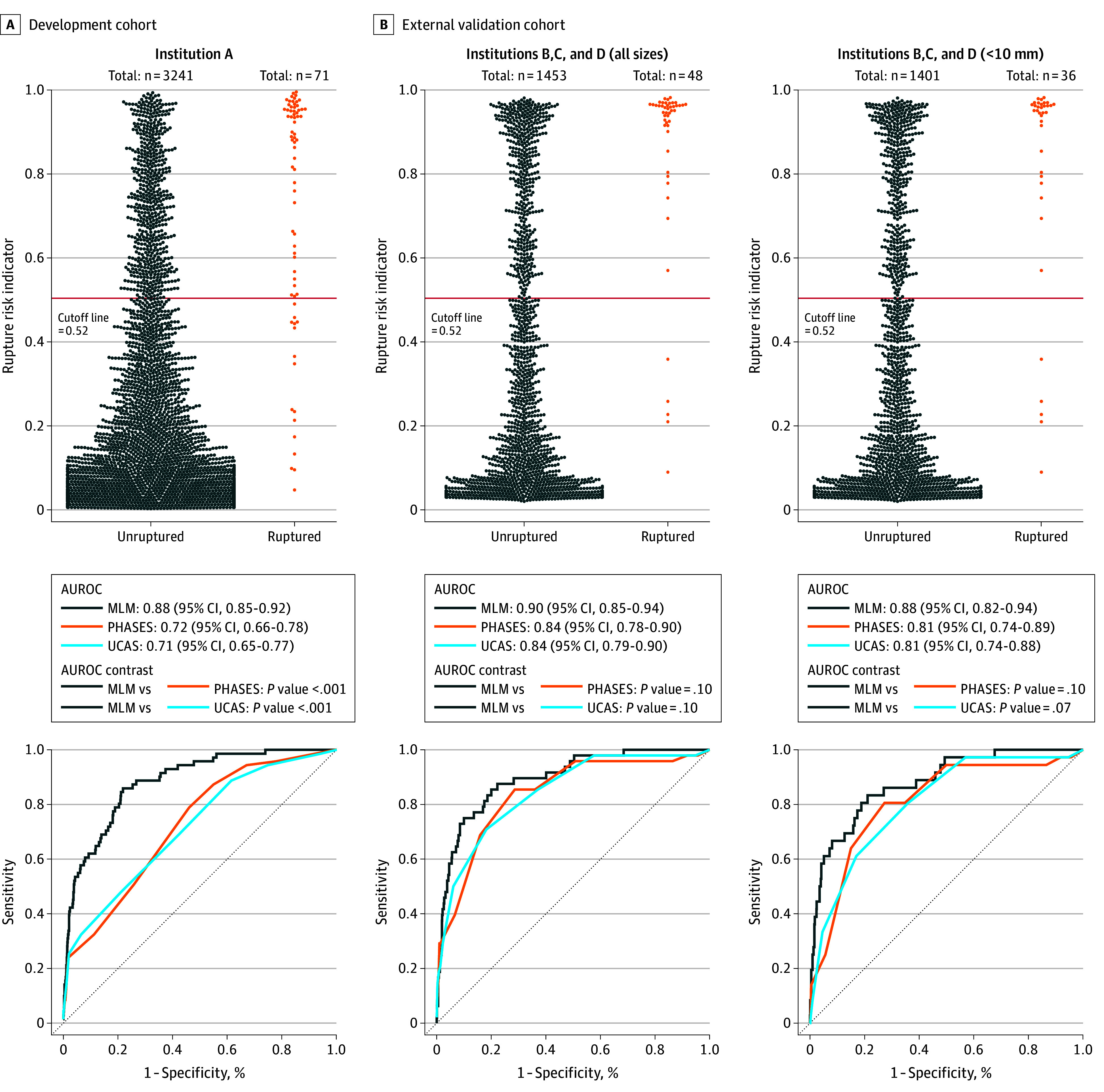
Development and External Validation of the Machine-Learning Model (MLM) and Receiver Operating Characteristic (ROC) Curves The rupture risk indicator is shown for the development (A) and external validation (B) cohorts. Each plot point represents an individual intracranial aneurysm. ROC curves are displayed for the MLM, PHASES (population, hypertension, age, size of aneurysm, earlier subarachnoid hemorrhage from another aneurysm, and site of aneurysm), and Unruptured Cerebral Aneurysm Study (UCAS), along with the corresponding area under the ROC (AUROC), values and 95% CIs. For PHASES, scores range from 0 to 2, with higher scores indicating greater predicted rupture risk; for the UCAS, scores range from 0 to 15, with higher scores indicating greater predicted rupture risk.

The rupture risk indicator from the developed MLM had a threshold of 0.52. Using this threshold, the sensitivity was 0.78 (95% CI, 0.67-0.86); specificity, 0.82 (95% CI, 0.80-0.83); PPV, 0.09 (95% CI, 0.07-0.11); NPV, 0.99 (95% CI, 0.99-1.00); PLR, 4.23 (95% CI, 3.66-4.89); NLR, 0.28 (95% CI, 0.18-0.42); and AUROC, 0.88 (95% CI, 0.85-0.92) for the prediction at institution A. Compared with MLM, the AUROC for PHASES was 0.72 (95% CI, 0.66-0.78; *P* < .001) and for the UCAS was 0.71 (95% CI, 0.65-0.77; *P* < .001) at institution A (Figure 2). Apparent calibration (intercept, −3.81 [95% CI, −4.19 to −3.51]; slope, 1.14 [95% CI, 0.94-1.38]; and Brier, 0.14 [95% CI, 0.13-0.15]) and logistic recalibration (intercept, 0 [95% CI, −0.59 to 0.54]; slope, 1.00 [95% CI, 0.82-1.21]; and Brier, 0.02 [95% CI, 0.01-0.02]) are shown in eFigure 2 in Supplement 1. The importance of the features in the developed MLM is shown in eFigure 3 in Supplement 1. The last aneurysm length during follow-up was considered the most significant feature associated with rupture risk. Eight of the top 10 most important features were associated with morphological parameters. Saccularity was the second-most important feature. Additionally, time-dependent morphological parameters were the fifth-most (for rate-length), sixth-most (for lr-length-b), and ninth-most (for lr-length-a) important features. As described previously, if no follow-up imaging was available, these time-dependent features were treated as missing, allowing the model to be applicable to aneurysms without longitudinal data. Additionally, the aneurysm location and age at diagnosis were ranked among the top 10 most important features. Among the features ranked between 11th and 20th, the PHASES score was 13th, and the UCAS scores was 18th. Clinical parameters, including history of hypertension, sex, and lifestyle factors, such as smoking and alcohol consumption, were ranked 21st and below. For further details, see eResults 2 in Supplement 1.

### External Validation Cohort and the Prediction Performance

For external validation, 1153 patients with 1501 UIAs with a median (IQR) follow-up of 5.4 (3.7-8.7) years from institutions B, C, and D were included (median [IQR] age, 63.6 [53.9-70.9] years; 828 females [71.8%] and 325 males [28.2%]). Of the 1501 UIAs, 48 (3.2%) ruptured during a median (IQR) follow-up of 5.4 (3.7-8.7) years. At institution B, 33 of 1035 UIAs (3.2%) in 804 patients had ruptured during 7255 aneurysm-years of follow-up (0.45% per year), with a median (IQR) follow-up of 6.4 (4.2-9.1) years. Similarly, at institution C, 4 of 265 UIAs (1.5%) in 206 patients had ruptured during 1036 aneurysm-years (0.39% per year) within a median (IQR) of 3.8 (3.0-4.6) years, and at institution D, 11 of 201 UIAs (5.5%) in 143 patients had ruptured during 1285 aneurysm-years (0.86% per year) within a median (IQR) of 5.7 (3.6-8.9) years (eFigure 1 in Supplement 1). The baseline characteristics of the external validation cohorts are shown in Table 2 and eTables 2-4 in Supplement 1. Additionally, 803 UIAs at institution B, 67 at institution C, and 50 at institution D each had 2 or more imaging measurements available.

**Table 2.** Patient and Aneurysm Characteristics in the Validation Cohort

Parameter	No. (%) or median (IQR)				*P* value^a^
	Patients, total (N = 1153)	Aneurysms			
		Total (N = 1501)	Ruptured (n = 48)	Unruptured (n = 1453)	
**Patient characteristics**					
Age, y	63.6 (53.9 to 70.9)	64.1 (54.2 to 71.0)	69.4 (60.6 to 75.2)	64 (54.1 to 70.9)	.006
Sex					
Female	828 (71.8)	1085 (72.3)	33 (68.8)	1052 (72.4)	.58
Male	325 (28.2)	416 (27.7)	15 (31.2)	401 (27.6)	
Multianeurysms	472 (40.9)	813 (54.2)	19 (39.6)	794 (54.6)	.04
No. of aneurysms	1 (1 to 2)	2 (1 to 2)	1 (1 to 2)	2 (1 to 2)	.008
Hypertension	523 (45.4)	698 (46.5)	29 (60.4)	669 (46.0)	.05
Alcohol consumption/wk	159 (38.9)	212 (39.2)	16 (44.4)	196 (38.8)	.51
Smoking	203 (17.6)	277 (18.5)	11 (22.9)	266 (18.3)	.42
No. of cigarettes/d	0 (0 to 15.0)	0 (0 to 15.0)	0 (0 to 11.0)	0 (0 to 15.0)	.64
y of Smoking	0 (0 to 20.0)	0 (0 to 23.0)	0 (0 to 24.2)	0 (0 to 22.0)	.90
Hyperlipidemia	235 (20.4)	301 (20.1)	6 (12.5)	295 (20.3)	.19
Diabetes	91 (7.9)	106 (7.1)	8 (16.7)	98 (6.7)	.008
SAH	53 (4.6)	76 (5.1)	5 (10.4)	71 (4.9)	.09
Polycystic kidney disease	12 (1.0)	17 (1.1)	1 (2.1)	16 (1.1)	.53
Cerebral hemorrhage	17 (1.5)	19 (1.3)	2 (4.2)	17 (1.2)	.07
Cerebral infarction	48 (4.2)	64 (4.3)	5 (10.4)	59 (4.1)	.03
Family history of a SAH	142 (12.3)	192 (12.8)	4 (8.3)	188 (12.9)	.35
Family history of an aneurysm	53 (4.6)	71 (4.7)	2 (4.2)	69 (4.7)	.85
Family history of a polycystic kidney	4 (0.3)	2 (0.3)	0 (0)	2 (0.2)	.72
mRS (first visit)	0	0	0 (0 to 1.0)	0	.01
Ethnicity^b^					
Japanese	805 (69.8)	1036 (69.0)	33 (68.8)	1003 (69.0)	.97
Other^c^	348 (30.2)	465 (31.0)	15 (31.2)	450 (31.0)	
**Aneurysm characteristics**					
Location					
ICA (all sites)	NA	761 (50.7)	11 (22.9)	750 (51.6)	<.001
ICA-Pcom	NA	141 (9.4)	8 (16.7)	133 (9.2)	.08
MCA (all sites)	NA	355 (23.7)	4 (8.3)	351 (24.2)	.01
ACA (all sites)	NA	233 (15.5)	18 (37.5)	215 (14.8)	<.001
ACA-Acom	NA	148 (9.9)	15 (31.3)	133 (9.2)	<.001
BA (all sites)	NA	103 (6.9)	13 (27.1)	90 (6.2)	<.001
BA-tip	NA	28 (1.9)	5 (10.4)	23 (1.6)	<.001
VA	NA	49 (3.3)	2 (4.2)	47 (3.2)	.72
Morphological parameters					
Presence of irregularity	NA	189 (12.7)	26 (55.3)	163 (11.3)	<.001
Length, mm	NA	2.5 (1.7 to 3.6)	6.3 (4.0 to 9.2)	2.5 (1.7 to 3.5)	<.001
Width, mm	NA	3.1 (2.3 to 4.4)	6.4 (4.6 to 9.3)	3.1 (2.3 to 4.3)	<.001
Neck diameter, mm	NA	2.8 (2.2 to 3.6)	4.0 (3.1 to 5.0)	2.8 (2.1 to 3.6)	<.001
Aspect ratio	NA	0.81 (0.63 to 1.00)	0.92 (0.78 to 1.17)	0.81 (0.63 to 1.00)	<.001
Saccularity, mm	NA	2.7 (1.7 to 4.6)	9.4 (5.1 to 19.2)	2.7 (1.7 to 4.4)	<.001
Time-dependent morphological parameters					
Rate-length, mm/d	NA	5.2 × 10^−5^ (−9.8 × 10^−5^ to 3.3 × 10^−4^)	1.6 × 10^−3^ (6.3 × 10^−4^ to 3.4 × 10^−3^)	4.5 × 10^−5^ (−1.0 × 10^−4^ to 2.9 × 10^−4^)	<.001
Rate-width, mmd/	NA	2.7 × 10^−5^ (−1.4 × 10^−4^ to 3.4 × 10^−4^)	1.4 × 10^−3^ (4.8 × 10^−4^ to 4.0 × 10^−3^)	0 (−1.4 × 10^−4^ to −3.1 × 10^−4^)	<.001
Rate-neck, mm/d	NA	0 (−2.1 × 10^−4^ to 2.5 × 10^−4^)	8.5 × 10^−4^ (1.5 × 10^−4^ to 1.7 × 10^−3^)	0 (−2.2 × 10^−4^ to −2.3 × 10^−4^)	<.001
lr-length-a	NA	4.0 × 10^−2^ (2.8 × 10^−2^ to 5.6 × 10^−2^)	7.4 × 10^−2^ (5.2 × 10^−2^ to 1.1 × 10^−1^)	3.9 × 10^−2^ (2.7 × 10^−2^ to 5.5 × 10^−2^)	<.001
lr-length-b	NA	1.0 × 10^−4^ (−1.5 × 10^−3^ to 2.3 × 10^−3^)	−9.1 × 10^−3^ (−2.0 × 10^−2^ to −7.0 × 10^−4^)	2.0 × 10^−4^ (−1.3 × 10^−3^ to 2.4 × 10^−3^)	<.001
lr-length-R	NA	0.99 (0.98 to 1.00)	0.98 (0.86 to 0.99)	0.99 (0.98 to 1.00)	.001
lr-width-a	NA	5.3 × 10^−2^ (3.9 × 10^−2^ to 7.4 × 10^−2^)	7.9 × 10^−2^ (6.3 × 10^−2^ to 1.2 × 10^−1^)	5.2 × 10^−2^ (3.9 × 10^−2^ to 7.3 × 10^−2^)	<.001
lr-width-b	NA	4.0 × 10^−4^ (−1.0 × 10^−3^ to 3.6 × 10^−3^)	−6.0 × 10^−3^ (−1.3 × 10^−2^ to −9.8 × 10^−4^)	5.0 × 10^−4^ (−8.5 × 10^−4^ to 3.7 × 10^−3^)	<.001
lr-width-R	NA	0.99 (0.98 to 1.00)	0.97 (0.92 to 0.99)	0.99 (0.98 to 1.00)	<.001
lr-neck-a	NA	4.8 × 10^−2^ (3.8 × 10^−2^ to 6.4 × 10^−2^)	6.0 × 10^−2^ (4.8 × 10^−2^ to 7.8 × 10^−2^)	4.8 × 10^−2^ (3.8 × 10^−2^ to 6.4 × 10^−2^)	.01
lr-neck-b	NA	9.0 × 10^−4^ (−4.0 × 10^−4^ to 4.8 × 10^−3^)	−9.0 × 10^−4^ (−4.9 × 10^−3^ to 1.2 × 10^−3^)	9.0 × 10^−4^ (−3.0 × 10^−4^ to 4.9 × 10^−3^)	<.001
lr-neck-R	NA	0.99 (0.98 to 1.00)	0.99 (0.96 to 1.00)	0.99 (0.98 to 1.00)	.04

Abbreviations: a, *a* parameter; ACA, anterior cerebral artery; Acom, anterior communicating artery; b, *b* parameter; BA, basilar artery; ICA, internal carotid artery; lr, *y* = *ax + b*; MCA, middle cerebral artery; mRS, modified Rankin scale; NA, not applicable; Pcom, posterior communicating artery; R, *R^2^* parameter; SAH, subarachnoid hemorrhage; VA, vertebral artery.

^a^ Clinical and morphological differences between ruptured and unruptured aneurysms were compared using the Fisher exact test for categorical variables and the Mann-Whitney test for continuous variables.

^b^ Categories were investigator-defined as Finnish, Japanese, or other based on prior evidence from the PHASES (population, hypertension, age, size of aneurysm, earlier SAH from another aneurysm, and site of aneurysm) score, but no Finnish patients were included.

^c^ Includes all patients not classified as Finnish or Japanese.

For the external validation cohort, the MLM was applied to predict rupture, demonstrating a sensitivity of 0.90 (95% CI, 0.78-0.95); specificity, 0.70 (95% CI, 0.67-0.72); PPV, 0.09 (95% CI, 0.07-0.12); NPV, 1.00 (95% CI, 0.99-1.00); PLR, 2.94 (95% CI, 2.60-3.33); NLR, 0.15 (95% CI, 0.07-0.34); and AUROC, 0.90 (95% CI, 0.85-0.94). All ruptured aneurysms measuring 4 mm were correctly identified by the model. Compared with MLM, PHASES had an AUROC value of 0.84 (95% CI, 0.78-0.90; *P* = .10) and the UCAS had an AUROC value of 0.84 (95% CI, 0.79-0.90; *P* = .10). Calibration with the fixed calibrator yielded an intercept of −1.14 (95% CI, −1.59 to −0.71), a slope of 0.85 (95% CI, 0.65-1.23), and a Brier score of 0.03 (95% CI, 0.03-0.04) (eFigure 4 in Supplement 1). Additionally, when applied to aneurysms less than 10 mm, the MLM had a sensitivity 0.86 (95% CI, 0.71-0.94); specificity, 0.71 (95% CI, 0.68-0.73); PPV, 0.07 (95% CI, 0.05-0.09); NPV, 1.00 (95% CI, 0.99-1.00); PLR, 2.94 (95% CI, 2.52-3.43); NLR, 0.20 (95% CI, 0.09-0.44); and AUROC, 0.88 (95% CI, 0.82-0.94). Compared with MLM, PHASES had an AUROC value applied to these smaller aneurysms of 0.81 (95% CI, 0.74-0.89; *P* = .10) and the UCAS had an AUROC value of 0.81 (95% CI, 0.74-0.88; *P* = .07). The external validation results included a comparison of each parameter between the true-positive and false-negative aneurysms (eTable 5 in Supplement 1). Additionally, differences in morphological parameters and imaging practices among the institutions are discussed in eResults 3 and eTables 6 and 7 in Supplement 1. Angiographic images and clinical data for false-negative aneurysms are shown in eFigures 5, 6, 7, 8, and 9 in Supplement 1, and additional details are provided in eResults 4 in Supplement 1.

## Discussion

This large, multicenter-cohort prognostic study developed an MLM to predict the rupture risk of UIAs using prerupture data. We achieved robust sensitivity and specificity in internal and external validations, drawing on more than 10 000 aneurysms from multiple institutions. A notable feature is the incorporation of established risk indices, such as PHASES and the UCAS, into the MLM’s input variables, effectively using widely recognized clinical indicators. Including time-dependent morphological parameters allowed the model to consider growth and morphological changes as risk factors, which is a feature of substantial clinical importance. Furthermore, the final model maintained robust performance across multiple cohorts, implying adaptability to different patient populations and imaging protocols. Interestingly, morphological factors, such as the most recent aneurysm length, saccularity, and aspect ratio, were highly ranked, supporting the widely held view that aneurysm geometry is of paramount importance in association with rupture risk.

Previous studies on rupture risk, determined by PHASES and the UCAS, have used multivariate logistic regression or survival analyses to identify factors independently associated with rupture.^18,20^ Although PHASES and the UCAS are straightforward and widely adopted tools, they do not fully capture the complexity of rupture dynamics. Previous studies have used MLMs to estimate rupture risk.^26,27^ However, many studies relied on data from already-ruptured aneurysms, raising concerns about the influence of prerupture and postrupture morphological changes on risk estimates. An aneurysm’s height, volume, and aspect ratio may change substantially after rupture, and 1 study showed an average 13.4% increase in height when comparing prerupture and postrupture measurements.^28^

The findings of the current study suggest that the aneurysm geometry remains a primary factor associated with rupture risk. Although previous studies have highlighted the importance of size,^18,20^ we showed that saccularity and time-dependent morphological changes were significantly associated with rupture risk. The significant role of the latest aneurysm length in the model is consistent with clinical observations, which highlight the importance of monitoring morphological changes.^20,29,30,31,32,33^ Regarding clinical variables, age, hypertension, a previous SAH, and the aneurysm location, each was associated with rupture risk. This finding is consistent with earlier studies and scoring models that acknowledged demographic and historical factors.^9,10,20,21,22,34,35^ Furthermore, although comparisons of the MLM with PHASES and the UCAS revealed no statistically significant difference in the AUROC in the development cohort, the MLM tended to have numerically higher performance values, suggesting its potential as a comprehensive predictive model that extends and enhances traditional risk factors. Conversely, aneurysms incorrectly predicted as nonruptured were typically small (≤3 mm), with limited follow-up scans or negligible size changes. Such aneurysms involve comorbid genetic disorders, such as sickle cell disease, suggesting that their rupture pathways may not be fully reflected in the training data used to build our model.^36^

### Strengths and Limitations

The strength of this study lies in its large-scale dataset sourced from multiple institutions, capturing a wide range of patient backgrounds. External validation across 3 independent international cohorts suggest that the model’s predictions are generalizable across institutions and regions. Additionally, the MLM incorporated morphological parameters that evolved with follow-up imaging while defaulting to baseline measurements when follow-up data were unavailable, ensuring the applicability of the MLM from initial patient presentation to longer-term surveillance. This point differs from rupture risk models that account for growth, which can only be applied once growth has been confirmed.^37^ Furthermore, the model was trained on clinical scenarios involving aneurysms that eventually ruptured, reflecting a decision-making process closely aligned with clinical settings.

This study also has some limitations. First, the model is designed to predict rupture within 2 years and may not directly apply to lifetime risk. The development of a longer-term model requires extensive, multiyear data, which are challenging to collect. Nevertheless, this does not mean that follow-up was restricted to 2 years. Patients were observed for longer periods (median [IQR] follow-up of 8.5 [5.1-11.6] years in the development cohort and 5.4 [3.7-8.7] years in the validation cohort), and all imaging data up to the last follow-up were used, while the prediction horizon was set at 2 years. Second, this study, to our knowledge, included more ruptured cases than similar studies.^18,20^ However, the rarity of rupture imposes inherent limitations on the analytic capacity of the study. Third, although calibration was assessed, some degree of miscalibration remained, which may limit direct probability interpretation in clinical practice. Fourth, imaging intervals and modalities, such as DSA, CTA, and MRA, were not standardized across institutions, which can introduce measurement variability. Although our model can incorporate time-dependent growth parameters, the precision of growth-related metrics may be affected by institution-specific scan intervals and modalities used. Additionally, rupture rates varied across institutions, reflecting differences in criteria for conservative management. Such heterogeneity may contribute to enhancing the external validity of the model. Furthermore, some studies have shown that more than 30% of patients with UIAs experience psychologic stress associated with chronic diseases, but such individual stress levels were not taken into account.^38,39,40,41^ Additionally, the model was primarily developed using a Japanese cohort and may not adequately account for racial and ethnic differences. The proportion of the internal carotid artery location in our cohorts was higher than in population-based studies, likely reflecting incidental detection and referral patterns, and this should be considered when interpreting generalizability.^42,43^ However, its consistency in external validation among several populations outside of Japan is promising. Additionally, traditional multivariate regression was not performed to define independent predictors.

The findings underscore the potential of MLM as a viable tool for assessing rupture risk, potentially opening a new frontier in aneurysm management. By integrating conventional scoring systems, such as PHASES and the UCAS, with an MLM that is able to handle multidimensional factors, including morphological changes, the model may improve risk stratification and guide more precise intervention decisions, particularly for UIAs less than 10 mm. The analytic code is not publicly available, but the model will be made available online as a web-based tool to estimate rupture risk of individual UIAs. Future studies should establish larger prospective international cohorts, standardize imaging intervals, and consider psychosocial or genetic factors to further refine risk estimates. The MLM-based risk indicator for UIAs, which integrates established indices and novel analysis, may support informed treatment decisions and advanced precision-medicine approaches tailored to patient-specific profiles.

## Conclusions

In this prognostic study, an MLM was developed and validated to predict the risk of UIA rupture in multiple international cohorts. The MLM demonstrated robust performance even for UIAs less than 10 mm and consistently identified features of UIAs as significantly associated with rupture across different cohorts with varied imaging protocols and clinical backgrounds. By incorporating morphological and clinical parameters, the model provided refined rupture risk assessment and may support individualized management and treatment decisions for physicians and patients.

## Notes

Supplement 1. **eMethods 1.** Data Collection **eMethods 2.** Development of MLM and External Validation Design **eResults 1.** Patient Characteristics **eResults 2.** Development Cohort and the MLM **eResults 3.** Differences Between Institutions A, B, C, and D **eResults 4.** Details of False-Negative Cases in the External Validation Cohort **eTable 1.** Patient and Aneurysm Characteristics of All Cohorts **eTable 2.** Patient and Aneurysm Characteristics in Institution B **eTable 3.** Patient and Aneurysm Characteristics in Institution C **eTable 4.** Patient and Aneurysm Characteristics in Institution D **eTable 5.** Difference Between True Positive and False Negative **eTable 6.** Difference of the Parameters Between Institutions A, B, C, and D **eTable 7.** Comparison of Medical Imaging Between Institutions A, B, C, and D **eFigure 1.** Probability of Aneurysm Rupture **eFigure 2.** Calibration Plot in the Development Cohort **eFigure 3.** Feature Importance of the Developed MLM **eFigure 4.** Calibration Plot in the External Validation Cohort **eFigure 5.** Case Summary of False-Negative Case 1 in Institution C **eFigure 6.** Case Summary of False-Negative Case 2 in Institution B **eFigure 7.** Case Summary of False-Negative Case 3 in Institution B **eFigure 8.** Case Summary of False-Negative Case 4 in Institution B **eFigure 9.** Case Summary of False-Negative Case 5 in Institution B

Supplement 2.
Data Sharing Statement
