# Supplementary material for: Development and Validation of a Prediction Model for Intracranial Aneurysm Rupture Risk
Source: JAMA Netw Open. 2025 Dec 23;8(12):e2550772. doi: 10.1001/jamanetworkopen.2025.50772 (PMC12728650; doi:10.1001/jamanetworkopen.2025.50772)
Supplement: Supplement 1. — eMethods 1. Data Collection eMethods 2. Development of MLM and External Validation Design eResults 1. Patient Characteristics eResults 2. Development Cohort and the MLM eResults 3. Differences Between Institutions A, B, C, and D eResults 4. Details of False-Negative Cases in the External Validation Cohort eTable 1. Patient and Aneurysm Characteristics of All Cohorts eTable 2. Patient and Aneurysm Characteristics in Institution B eTable 3. Patient and Aneurysm Characteristics in Institution C eTable 4. Patient and Aneurysm Characteristics in Institution D eTable 5. Difference Between True Positive and False Negative eTable 6. Difference of the Parameters Between Institutions A, B, C, and D eTable 7. Comparison of Medical Imaging Between Institutions A, B, C, and D eFigure 1. Probability of Aneurysm Rupture eFigure 2. Calibration Plot in the Development Cohort eFigure 3. Feature Importance of the Developed MLM eFigure 4. Calibration Plot in the External Validation Cohort eFigure 5. Case Summary of False-Negative Case 1 in Institution C eFigure 6. Case Summary of False-Negative Case 2 in Institution B eFigure 7. Case Summary of False-Negative Case 3 in Institution B eFigure 8. Case Summary of False-Negative Case 4 in Institution B eFigure 9. Case Summary of False-Negative Case 5 in Institution B [file jamanetwopen-e2550772-s001.pdf]

Supplemental Online Content

Fujimura S, Yanagisawa T, Kudo G, et al. Development and validation of a prediction model for intracranial aneurysm rupture risk. *JAMA Netw Open*. 2025;8(12):e2550772. doi:10.1001/jamanetworkopen.2025.50772

eMethods 1. Data Collection
eMethods 2. Development of MLM and External Validation Design
eResults 1. Patient Characteristics
eResults 2. Development Cohort and the MLM
eResults 3. Differences Between Institutions A, B, C, and D
eResults 4. Details of False-Negative Cases in the External Validation Cohort
eTable 1. Patient and Aneurysm Characteristics of All Cohorts
eTable 2. Patient and Aneurysm Characteristics in Institution B
eTable 3. Patient and Aneurysm Characteristics in Institution C
eTable 4. Patient and Aneurysm Characteristics in Institution D
eTable 5. Difference Between True Positive and False Negative
eTable 6. Difference of the Parameters Between Institutions A, B, C, and D
eTable 7. Comparison of Medical Imaging Between Institutions A, B, C, and D
eFigure 1. Probability of Aneurysm Rupture
eFigure 2. Calibration Plot in the Development Cohort
eFigure 3. Feature Importance of the Developed MLM
eFigure 4. Calibration Plot in the External Validation Cohort
eFigure 5. Case Summary of False-Negative Case 1 in Institution C
eFigure 6. Case Summary of False-Negative Case 2 in Institution B
eFigure 7. Case Summary of False-Negative Case 3 in Institution B
eFigure 8. Case Summary of False-Negative Case 4 in Institution B
eFigure 9. Case Summary of False-Negative Case 5 in Institution B

This supplemental material has been provided by the authors to give readers additional information about their work.

© 2025 Fujimura S et al. *JAMA Network Open*.

eMethods 1. Data Collection

Patients and/or aneurysms for which these outcomes could not be evaluated or confirmed due to (1) aneurysm-unrelated death, (2) cessation of follow-up, (3) undetectable aneurysms on repeated imaging, (4) unclear aneurysm details, (5) previous treatment at another institution, (6) transfer to another institution during follow-up, (7) one-time consultation as a second opinion only, or (8) loss to follow-up or incomplete records were excluded.

Detailed patient characteristics collected included number of cigarettes per day, smoking duration in years, history of hyperlipidemia, diabetes, subarachnoid hemorrhage, polycystic kidney disease, cerebral hemorrhage, and cerebral infarction; family history of subarachnoid hemorrhage, unruptured intracranial aneurysm, and polycystic kidney disease; and modified Rankin scale score at initial diagnosis.

Aneurysm location was further subdivided as follows: ICA (posterior communicating segment and others), ACA (anterior communicating artery and others), BA (tip and others, including the superior cerebellar artery, anterior inferior cerebellar artery, and posterior cerebral artery), and VA (including the posterior inferior cerebellar artery).

eMethods 2. Development of MLM and External Validation Design

LightGBM was chosen for its ability to handle high-dimensional nonlinear tabular data, process missing values without imputation, and provide fast, memory-efficient training suitable for large multicenter datasets. Because ruptured cases were the minority, we undersampled the unruptured cases. To avoid loss of data diversity inherent in simple random undersampling, k-means clustering was applied to divide the unruptured cases into several clusters, from which samples were evenly removed to improve class balance while preserving dataset characteristics. Given the reduced training size after undersampling, semisupervized learning was introduced with treated aneurysms. An MLM trained on undersampled data was applied to the treated set, identifying five aneurysms with the highest rupture risk indicators (0.90–0.95) as pseudoruptured. This procedure was repeated until 120 pseudoruptured aneurysms were selected. The pseudoruptured aneurysms were analyzed with the actual ruptured aneurysms, whereas unruptured aneurysms were undersampled to match the 191 ruptured aneurysms, yielding 382 datasets. A total of 40 MLMs were created through 10 stratified quadrant cross-validations, and their mean rupture risk indicator was used. A threshold for classifying ruptured against unruptured aneurysms was obtained by finding the receiver operating characteristic curve point minimizing Euclidean distance to the ideal (true-positive rate = 1; false-positive rate = 0). The average threshold across the 40 models was applied to external data.

© 2025 Fujimura S et al. *JAMA Network Open*.

eResults 1. Patient Characteristics

The median age of patients with ruptured aneurysms was 67.4 years, and 27.7% were male. The median age of patients with unruptured aneurysms was 65.3 years, and 30.2% were male. Additionally, 11.8% of patients with ruptured aneurysms had a history of SAH (P <.001), and the proportion of patients with hypertension was higher in the ruptured group (25.2%) than in the unruptured group (17.5%) (P = .18).

eResults 2. Development Cohort and the MLM

Notably, in eFigure 3, the importance presented indicates only the relative contribution of each parameter to the predictions and does not reflect the absolute importance or specific tendency toward rupture risk. For aneurysms with a single imaging measurement available, the “last” measurement coincided with the baseline length. The width and neck diameter were the third and eighth important features, respectively. The AR, which was frequently associated with rupture risk in previous studies, was the seventh most important feature. Eight of the features ranked between the 11th and 20th were time-dependent morphological parameters.

eResults 3. Differences Between Institutions A, B, C, and D

eTable 6 shows a comparison of variables across institutions. The results showed statistically significant differences in many morphological parameters. Among the four institutions, the largest average aneurysm length was observed at Institution-D (3.5 mm), followed by Institution-A (2.8 mm), Institution-C (2.7 mm), and Institution-B (2.4 mm). Notably, Institution-D exhibited greater values than the other institutions in several morphological parameters, including rate-length (Institution-D: 1.85×10^{-4}, Institution-B: 4.88×10^{-5}, Institution-A: 2.10×10^{-5}, and Institution-C: 0), saccularity (Institution-D: 4.7, Institution-A: 3.4, Institution-C: 2.7, and Institution-B: 2.6), and aspect ratio (AR) (Institution-D: 1.1, Institution-A: 1.0, Institution-C: 0.9, and Institution-B: 0.7). eTable 7 shows a comparison of imaging practices among Institutions-A, B, C, and D. At Institutions-A, B, and C, CTA or DSA was used for diagnosing 91.5%, 78.8%, and 100% of ruptured aneurysms, respectively, at least at the initial and final imaging stages. For unruptured aneurysms, the percentages were 86.7%, 88.3%, and 79.5%, respectively. Conversely, Institution-D had a CTA or DSA usage rate of 90.9% for ruptured aneurysms and only 45.5% for unruptured aneurysms. Overall, more than half (52.0%) of the aneurysms at Institution-D were imaged using MRA at both the initial and final stages. The mean number of imaging sessions during follow-up across all modalities was 5.3 ± 4.3 for all institutions (Institution-A: 6.2 ± 4.7, Institution-B: 3.8 ± 2.1, Institution-C: 2.0 ± 0.7, and Institution-D: 2.2 ± 1.0). Institution-A had the most frequent imaging intervals, with ruptured aneurysms diagnosed approximately every 6–12 months (7.7 ± 6.9 months) and unruptured aneurysms diagnosed every 12–24 months (13.7 ± 10.3 months). Conversely, Institution-D, which had the longest intervals between imaging examinations, conducted imaging approximately once every 12–18 months (13.3 ± 6.4 months) for ruptured aneurysms and once every 60 months (60.3 ± 37.5 months) for unruptured aneurysms.

© 2025 Fujimura S et al. *JAMA Network Open.*

eResults 4. Details of False-Negative Cases in the External Validation Cohort

In one false-negative aneurysm at Institution-C, time-dependent morphological parameters could not be assessed due to the availability of only one imaging examination before rupture (eFigure 5). The aneurysm, measured 19 months before rupture, was 3 mm in length and 2.5 mm in width and had a neck diameter of 3.1 mm. The patient had sickle cell disease but no major genetic disorders related to aneurysms. The four false-negative aneurysms at Institution-B were less than 3 mm in length (median, 1.9 mm) before rupture (eFigure 6–9). Except for one aneurysm in the MCA (1.3 mm in length, 2.3 mm in width, and 2.6 mm neck diameter) (eFigure 6), multiple imaging examinations were available for all aneurysms. However, the size changes before rupture were minimal.

eTable 1. Patient and Aneurysm Characteristics of All Cohorts

Parameter	Patients	Aneurysms			P Value[a]
	Total	Total	Ruptured	Unruptured	
	Number (%) or Median (IQR)	Number (%) or Median (IQR)	Number (%) or Median (IQR)	Number (%) or Median (IQR)	
	N = 3903	N = 4813	N = 119	N = 4694	
**Patient Characteristics**					
Age	64.8 (54.4–72.5)	65.4 (55.1–72.9)	67.4 (54.3–74.3)	65.3 (55.1–72.8)	.28
Sex (male)	1219 (31.2%)	1451 (30.1%)	33 (27.7%)	1418 (30.2%)	.56
Multianeurysms	1289 (33.0%)	2192 (45.5%)	49 (41.2%)	2143 (45.7%)	.33
Number of Aneurysms	1 (1–2)	1 (1–2)	1 (1–2)	1 (1–2)	.22
Hypertension	1727 (44.2%)	682 (17.8%)	27 (25.2%)	655 (17.5%)	.18
Alcohol consumption per week	562 (14.4%)	682 (17.8%)	27 (25.2%)	655 (17.5%)	.04
Smoking	575 (14.7%)	726 (15.1%)	19 (16.0%)	707 (15.1%)	.79
Number of cigarettes per day	0 (0–10)	0 (0–10)	0 (0–10)	0 (0–10)	.41
Years of smoking	0 (0–10)	0 (0–10)	0 (0–6)	0 (0–10)	.73
Hyperlipidemia	715 (18.3%)	878 (18.2%)	14 (11.8%)	864 (18.4%)	.06
Diabetes mellitus	269 (6.9%)	325 (6.8%)	10 (8.4%)	315 (6.7%)	.47
SAH	123 (3.2%)	158 (3.3%)	14 (11.8%)	144 (3.1%)	<.001
Polycystic kidney disease	52 (1.3%)	63 (1.3%)	3 (2.5%)	60 (1.3%)	.24
Cerebral hemorrhage	22 (0.6%)	25 (0.5%)	3 (2.5%)	22 (0.5%)	.002
Cerebral infarction	52 (1.3%)	69 (1.4%)	5 (4.2%)	64 (1.4%)	.01
Family history of SAH	480 (12.3%)	608 (12.6%)	14 (11.8%)	594 (12.7%)	.77
Family history of an aneurysm	116 (3.0%)	148 (3.1%)	4 (3.4%)	144 (3.1%)	.86
Family history of a polycystic kidney	16 (0.4%)	17 (0.4%)	0 (0.0%)	17 (0.4%)	.51
mRS (first visit)	0 (0–0)	0 (0–0)	0 (0–0)	0 (0–0)	.007
Race/ethnicity (Japanese)[b]	3531 (90.5%)	4320 (89.8%)	103 (86.6%)	4217 (89.8%)	.24

© 2025 Fujimura S et al. *JAMA Network Open.*

eTable 1. Patient and Aneurysm Characteristics of All Cohorts (continued)

**Aneurysm Characteristics**					
**Location**					
ICA (all sites)		2624 (54.5%)	36 (30.3%)	2588 (55.1%)	<.001
ICA-Pcom		744 (15.5%)	26 (21.8%)	718 (15.3%)	.05
MCA (all sites)		1103 (22.9%)	20 (16.8%)	1083 (23.1%)	.11
ACA (all sites)		702 (14.6%)	35 (29.4%)	667 (14.2%)	<.001
ACA-Acom		458 (9.5%)	25 (21.0%)	433 (9.2%)	<.001
BA (all sites)		294 (6.1%)	24 (20.2%)	270 (5.8%)	<.001
BA-tip		108 (2.2%)	12 (10.1%)	96 (2.0%)	<.001
VA		90 (1.9%)	4 (3.4%)	86 (1.8%)	.22
**Morphological Parameters**					
Presence of irregularity		348 (7.3%)	50 (42.4%)	298 (6.4%)	<.001
Length		2.7 (2.0–3.7)	5.8 (3.6–8.1)	2.7 (2.0–3.6)	<.001
Width		3.3 (2.5–4.4)	5.3 (3.9–8.0)	3.3 (2.5–4.4)	<.001
Neck diameter		3.1 (2.5–3.9)	3.9 (3.0–5.2)	3.1 (2.5–3.9)	<.001
AR		0.90 (0.72–1.09)	0.99 (0.78–1.27)	0.90 (0.72–1.09)	.001
Saccularity		3.1 (2.1–4.7)	7.0 (4.3–12.6)	3.1 (2.1–4.6)	<.001

© 2025 Fujimura S et al. *JAMA Network Open*.

eTable 1. Patient and Aneurysm Characteristics of All Cohorts (continued)

**Aneurysm Characteristics**					
**Time-Dependent Morphological Parameters**					
Rate-length		2.8×10^{-5}<br>(-1.0×10^{-4}–2.5×10^{-4})	1.3×10^{-3}<br>(2.8×10^{-4}–3.7×10^{-3})	2.4×10^{-5}<br>(-1.1×10^{-4}–2.3×10^{-4})	<.001
Rate-width		4.4×10^{-5}<br>(-1.6×10^{-4}–3.2×10^{-4})	7.4×10^{-4}<br>(0–3.0×10^{-3})	3.8×10^{-5}<br>(-1.6×10^{-4}–3.1×10^{-4})	<.001
Rate-neck		6.7×10^{-7}<br>(-1.7×10^{-4}–2.4×10^{-4})	3.9×10^{-4}<br>(0–1.2×10^{-3})	0<br>(-1.7×10^{-4}–2.2×10^{-4})	<.001
lr-length-a		4.4×10^{-2}<br>(3.2×10^{-2}–6.0×10^{-2})	7.3×10^{-2}<br>(5.4×10^{-2}–9.7×10^{-2})	4.4×10^{-2}<br>(3.2×10^{-2}–5.9×10^{-2})	<.001
lr-length-b		4.0×10^{-4}<br>(-9.0×10^{-4}–3.0×10^{-3})	-1.9×10^{-3}<br>(-8.0×10^{-3}–-2.5×10^{-4})	4.0×10^{-4}<br>(-8.0×10^{-4}–3.1×10^{-3})	<.001
lr-length-R		0.99<br>(0.98–1.00)	0.99<br>(0.94–1.00)	0.99<br>(0.98–1.00)	.001
lr-width-a		5.4×10^{-2}<br>(4.2×10^{-2}–7.2×10^{-2})	7.8×10^{-2}<br>(6.1×10^{-2}–1.1×10^{-1})	5.3×10^{-2}<br>(4.1×10^{-2}–7.1×10^{-2})	<.001
lr-width-b		4.0×10^{-4}<br>(-1.1×10^{-3}–3.5×10^{-3})	-9.0×10^{-4}<br>(-6.2×10^{-3}–4.0×10^{-4})	4.0×10^{-4}<br>(-1.0×10^{-3}–3.6×10^{-3})	<.001
lr-width-R		0.99<br>(0.98–1.00)	0.99<br>(0.95–1.00)	0.99<br>(0.98–1.00)	.003
lr-neck-a		5.3×10^{-2}<br>(4.2×10^{-2}–6.5×10^{-2})	5.9×10^{-2}<br>(4.7×10^{-2}–7.9×10^{-2})	5.3×10^{-2}<br>(4.2×10^{-2}–6.5×10^{-2})	.005
lr-neck-b		8.0×10^{-4}<br>(-4.0×10^{-4}–4.4×10^{-3})	-2.0×10^{-3}<br>(-2.4×10^{-3}–7.0×10^{-4})	9.0×10^{-4}<br>(-3.0×10^{-4}–4.5×10^{-3})	<.001
lr-neck-R		0.99<br>(0.98–1.00)	0.99<br>(0.98–1.00)	0.99<br>(0.98–1.00)	.82

Abbreviations: ACA, anterior cerebral artery; Acom, anterior communicating artery; AR, aspect ratio; BA, basilar artery; ICA, internal carotid artery; IQR, interquartile range; MCA, middle cerebral artery; mRS, modified Rankin Scale; Pcom, posterior communicating artery; SAH, subarachnoid hemorrhage; VA, vertebral artery.

[a] Clinical and morphological differences between ruptured and unruptured aneurysms were compared using Fisher's exact test for categorical variables and the Mann–Whitney U test for continuous variables.

[b] Race/ethnicity categories were investigator defined as Finnish, Japanese, or Other based on prior evidence from the PHASES score. No Finnish patients were included; therefore, the final categories analyzed were Japanese and Other. "Other" includes all patients not classified as Finnish or Japanese.

© 2025 Fujimura S et al. *JAMA Network Open.*

eTable 2. Patient and Aneurysm Characteristics in Institution B

Parameter	Patients	Aneurysms			
	Total	Total	Ruptured	Unruptured	P Value[a]
	Number (%) or Median (IQR)	Number (%) or Median (IQR)	Number (%) or Median (IQR)	Number (%) or Median (IQR)	
	N = 804	N = 1035	N = 33	N = 1002	
**Patient Characteristics**					
Age	64.6 (55.0–71.5)	65.1 (55.5–71.7)	72 (67.0–79.0)	64.8 (55.2–71.5)	<.001
Sex (male)	238 (29.6%)	306 (29.6%)	9 (27.3%)	297 (29.6%)	.77
Multianeurysms	312 (38.8%)	543 (52.5%)	14 (42.4%)	529 (52.8%)	.24
Number of aneurysms	1 (1–2)	2 (1–2)	1 (1–2)	2 (1–2)	.22
Hypertension	324 (40.3%)	429 (41.4%)	21 (63.6%)	408 (40.7%)	.009
Alcohol consumption per week	60 (7.5%)	75 (24.7%)	6 (24.0%)	69 (24.7%)	.94
Smoking	123 (15.3%)	167 (16.1%)	5 (15.2%)	162 (16.2%)	.88
Number of cigarettes per day	0 (0–10)	0 (0–10)	0 (0–5)	0 (0–10)	.31
Years of smoking	0 (0–20)	0 (0–20)	0 (0–10)	0 (0–20)	.58
Hyperlipidemia	104 (12.9%)	129 (12.5%)	4 (12.1%)	125 (12.5%)	.95
Diabetes mellitus	58 (7.2%)	63 (6.1%)	7 (21.2%)	56 (5.6%)	<.001
SAH	8 (1.0%)	13 (1.3%)	1 (3.0%)	12 (1.2%)	.36
Polycystic kidney disease	8 (1.0%)	11 (1.1%)	1 (3.0%)	10 (1.0%)	.26
Cerebral hemorrhage	9 (1.1%)	9 (0.9%)	2 (6.1%)	7 (0.7%)	.001
Cerebral infarction	27 (3.4%)	34 (3.3%)	4 (12.1%)	30 (3.0%)	.004
Family history of SAH	93 (11.6%)	121 (11.7%)	4 (12.1%)	117 (11.7%)	.94
Family history of an aneurysm	13 (1.6%)	18 (1.7%)	0 (0%)	18 (1.8%)	.44
Family history of a polycystic kidney	2 (0.2%)	2 (0.2%)	0 (0%)	2 (0.2%)	.80
mRS (first visit)	0 (0–0)	0 (0–0)	0 (0–0)	0 (0–0)	<.001
Race/ethnicity (Japanese)[b]	804 (100%)	1035 (100%)	33 (100%)	1002 (100%)	1.00

© 2025 Fujimura S et al. *JAMA Network Open.*

eTable 2. Patient and Aneurysm Characteristics in Institution B (continued)

**Aneurysm Characteristics**					
**Location**					
ICA (all sites)		556 (53.7%)	8 (24.2%)	548 (54.7%)	.001
ICA-Pcom		105 (10.1%)	8 (24.2%)	97 (9.7%)	.007
MCA (all sites)		222 (21.4%)	2 (6.1%)	220 (22.0%)	.03
ACA (all sites)		158 (15.3%)	12 (36.4%)	146 (14.6%)	.001
ACA-Acom		107 (10.3%)	10 (30.3%)	97 (9.7%)	<.001
BA (all sites)		62 (6.0%)	9 (27.3%)	53 (5.3%)	<.001
BA-tip		28 (2.7%)	5 (15.2%)	23 (2.3%)	<.001
VA		37 (3.6%)	2 (6.1%)	35 (3.5%)	.44
**Morphological Parameters**					
Presence of irregularity		140 (13.6%)	19 (59.4%)	121 (12.1%)	<.001
Length		2.4 (1.6–3.4)	5.5 (4.0–7.5)	2.3 (1.6–3.2)	<.001
Width		3.2 (2.4–4.5)	6.0 (4.5–7.7)	3.2 (2.4–4.4)	<.001
Neck diameter		3.0 (2.4–3.8)	4.0 (3.3–5.1)	3.0 (2.3–3.8)	<.001
AR		0.73 (0.57–0.89)	0.90 (0.78–1.16)	0.72 (0.57–0.89)	<.001
Saccularity		2.6 (1.6–4.0)	8.4 (4.7–11.8)	2.5 (1.6–3.9)	<.001

© 2025 Fujimura S et al. *JAMA Network Open.*

eTable 2. Patient and Aneurysm Characteristics in Institution B (continued)

**Aneurysm Characteristics**					
**Time-Dependent Morphological Parameters**					
Rate-length		4.9×10^{-5} (-8.9×10^{-5}–3.1×10^{-4})	1.7×10^{-3} (8.7×10^{-4}–2.8×10^{-3})	4.5×10^{-5} (-9.3×10^{-5}–2.7×10^{-4})	<.001
Rate-width		2.7×10^{-5} (-1.4×10^{-4}–3.3×10^{-4})	1.2×10^{-3} (4.6×10^{-4}–4.4×10^{-3})	0 (-1.4×10^{-4}–3.1×10^{-4})	<.001
Rate-neck		0 (-1.8×10^{-4}–2.2×10^{-4})	9.1×10^{-4} (1.2×10^{-4}–1.7×10^{-3})	0 (-2.0×10^{-4}–2.1×10^{-4})	<.001
lr-length-a		3.8×10^{-2} (2.7×10^{-2}–5.5×10^{-2})	6.3×10^{-2} (5.0×10^{-2}–8.5×10^{-2})	3.8×10^{-2} (2.7×10^{-2}–5.3×10^{-2})	<.001
lr-length-b		1.0×10^{-4} 2.0 (-1.4×10^{-3}–2.2×10^{-3})	-7.8×10^{-3} (-2.3×10^{-2}–-9.3×10^{-4})	2.0×10^{-4} (-1.3×10^{-3}–2.3×10^{-3})	<.001
lr-length-R		0.99 (0.98–1.00)	0.989 (0.85–0.99)	0.99 (0.98–1.00)	<.001
lr-width-a		5.3×10^{-2} (3.9×10^{-2}–7.4×10^{-2})	7.6×10^{-2} (6.2×10^{-2}–9.6×10^{-2})	5.2×10^{-2} (3.9×10^{-2}–7.3×10^{-2})	<.001
lr-width-b		4.5×10^{-4} (-1.0×10^{-3}–3.4×10^{-3})	-5.3×10^{-3} (-1.0×10^{-2}–-9.3×10^{-4})	5.0×10^{-4} (-8.3×10^{-4}–3.6×10^{-3})	<.001
lr-width-R		0.99 (0.98–1.00)	0.97 (0.91–0.99)	1.00 (0.98–1.00)	<.001
lr-neck-a		4.9×10^{-2} (3.9×10^{-2}–6.5×10^{-2})	6.0×10^{-2} (4.7×10^{-2}–7.6×10^{-2})	4.9×10^{-2} (3.9×10^{-2}–6.5×10^{-2})	.05
lr-neck-b		9.0×10^{-4} (-3.0×10^{-4}–4.8×10^{-3})	-9.0×10^{-4} (-6.8×10^{-3}–1.3×10^{-3})	1.0×10^{-3} (-2.0×10^{-4}–-4.9×10^{-3})	<.001
lr-neck-R		0.99 (0.98–1.00)	0.99 (0.94–1.00)	0.99 (0.98–1.00)	.04

Abbreviations: ACA, anterior cerebral artery; Acom, anterior communicating artery; AR, aspect ratio; BA, basilar artery; ICA, internal carotid artery; IQR, interquartile range; MCA, middle cerebral artery; mRS, modified Rankin Scale; Pcom, posterior communicating artery; SAH, subarachnoid hemorrhage; VA, vertebral artery.

[a] Clinical and morphological differences between ruptured and unruptured aneurysms were compared using Fisher's exact test for categorical variables and the Mann–Whitney U test for continuous variables.

[b] Race/ethnicity categories were investigator defined as Finnish, Japanese, or Other based on prior evidence from the PHASES score. No Finnish patients were included; therefore, the final categories analyzed were Japanese and Other. "Other" includes all patients not classified as Finnish or Japanese.

© 2025 Fujimura S et al. *JAMA Network Open.*

eTable 3. Patient and Aneurysm Characteristics in Institution C

Parameter	Patients	Aneurysms			P Value[a]
	Total	Total	Ruptured	Unruptured	
	Number (%) or Median (IQR)	Number (%) or Median (IQR)	Number (%) or Median (IQR)	Number (%) or Median (IQR)	
	N = 206	N = 265	N = 4	N = 261	
**Patient Characteristics**					
Age	63.3 (52.8–72.7)	64 (54.1–72.7)	51.5 (37.6–66.2)	64.1 (54.3–72.7)	.31
Sex (male)	56 (27.2%)	64 (24.2%)	3 (75.0%)	61 (23.4%)	.02
Multianeurysms	110 (53.4%)	169 (63.8%)	3 (75.0%)	166 (63.6%)	.64
Number of aneurysms	2 (1–2)	2 (1–3)	2 (1.75–3)	2 (1–3)	.66
Hypertension	125 (60.7%)	168 (63.4%)	2 (50.0%)	166 (63.6%)	.58
Alcohol consumption per week	25 (12.1%)	29 (42.0%)	2 (100%)	27 (40.3%)	.10
Smoking	25 (12.1%)	31 (11.7%)	0 (0%)	31 (11.9%)	.47
Number of cigarettes per day	5 (0–20)	5 (0–20)	15 (7.5–20)	5 (0–20)	.51
Years of smoking	2 (0–30)	4 (0–30)	8.5 (1.5–16.25)	4 (0–30)	.95
Hyperlipidemia	103 (50.0%)	139 (52.5%)	2 (50.0%)	137 (52.5%)	.92
Diabetes mellitus	25 (12.1%)	34 (12.8%)	0 (0%)	34 (13.0%)	.44
SAH	27 (13.1%)	37 (14.0%)	2 (50.0%)	35 (13.4%)	.04
Polycystic kidney disease	1 (0.5%)	1 (0.4%)	0 (0%)	1 (0.4%)	.93
Cerebral hemorrhage	8 (3.9%)	10 (3.8%)	0 (0%)	10 (3.8%)	.70
Cerebral infarction	0 (0%)	0 (0%)	0 (0%)	0 (0%)	1.00
Family history of SAH	20 (9.7%)	30 (11.3%)	0 (0%)	30 (11.5%)	.48
Family history of an aneurysm	19 (9.2%)	25 (9.4%)	0 (0%)	25 (9.6%)	.52
Family history of a polycystic kidney	0 (0%)	0 (0%)	0 (0%)	0 (0%)	1.00
mRS (first visit)	0 (0–0)	0 (0–0)	2 (0.75–3)	0 (0–0)	.04
Race/ethnicity (Japanese)[b]	1 (0.5%)	1 (0.4%)	0 (0%)	1 (0.4%)	.93

© 2025 Fujimura S et al. *JAMA Network Open.*

eTable 3. Patient and Aneurysm Characteristics in Institution C (continued)

**Aneurysm Characteristics**					
**Location**					
ICA (all sites)		158 (59.6%)	2 (50.0%)	156 (59.8%)	.70
ICA-Pcom		25 (9.4%)	0 (0%)	25 (9.6%)	.52
MCA (all sites)		43 (16.2%)	1 (25.0%)	42 (16.1%)	.64
ACA (all sites)		38 (14.3%)	0 (0%)	38 (14.6%)	.41
ACA-Acom		22 (8.3%)	0 (0%)	22 (8.4%)	.55
BA (all sites)		21 (7.9%)	1 (25.0%)	20 (7.7%)	.21
BA-tip		0 (0%)	0 (0%)	0 (0%)	1.00
VA		5 (1.9%)	0 (0%)	5 (1.9%)	.79
**Morphological Parameters**					
Presence of irregularity		15 (5.7%)	1 (25.0%)	14 (5.4%)	.10
Length		2.7 (1.9–3.8)	5.2 (3.5–7.3)	2.6 (1.9–3.8)	.02
Width		2.8 (2.0–3.8)	6.4 (3.9–8.7)	2.8 (2.0–3.8)	.04
Neck diameter		2.5 (2.0–3.3)	3.9 (3.0–5.2)	2.5 (2.0–3.3)	.05
AR		0.93 (0.82–1.09)	0.95 (0.79–1.14)	0.93 (0.82–1.09)	.97
Saccularity		2.7 (1.6–4.7)	7.6 (5.2–10.9)	2.7 (1.6–4.7)	.04

© 2025 Fujimura S et al. *JAMA Network Open*.

eTable 3. Patient and Aneurysm Characteristics in Institution C (continued)

**Aneurysm Characteristics**					
**Time-Dependent Morphological Parameters**					
Rate-length		0 (-3.6×10^{-4}–5.5×10^{-4})	5.6×10^{-3} (5.4×10^{-4}–5.8×10^{-4})	0 (-3.9×10^{-4}–4.9×10^{-4})	.22
Rate-width		0 (-2.3×10^{-4}–5.6×10^{-4})	2.5×10^{-3} (2.4×10^{-3}–2.6×10^{-3})	0 (-2.6×10^{-4}–4.7×10^{-4})	.04
Rate-neck		0 (-3.0×10^{-4}–5.9×10^{-4})	3.5×10^{-2} (2.3×10^{-3}–4.6×10^{-3})	0 (-3.1×10^{-4}–5.4×10^{-4})	.04
lr-length-a		4.6×10^{-2} (3.1×10^{-2}–5.5×10^{-2})	1.1×10^{-1} (1.1×10^{-1}–1.1×10^{-1})	4.4×10^{-2} (3.0×10^{-2}–5.4×10^{-2})	.03
lr-length-b		3.0×10^{-4} (-7.0×10^{-4}–3.6×10^{-3})	-2.5×10^{-4} (-3.3×10^{-4}–-1.8×10^{-4})	4.0×10^{-4} (-8.0×10^{-4}–4.0×10^{-3})	.44
lr-length-R		0.99 (0.97–1.00)	1.00 (1.00–1.00)	0.99 (0.97–1.00)	.03
lr-width-a		4.9×10^{-2} (3.4×10^{-2}–6.3×10^{-2})	1.3×10^{-1} (1.1×10^{-1}–1.4×10^{-1})	4.7×10^{-2} (3.4×10^{-2}–6.1×10^{-2})	.03
lr-width-b		1.0×10^{-4} (-1.3×10^{-3}–2.3×10^{-3})	-2.0×10^{-3} (-2.5×10^{-3}–-1.4×10^{-3})	1.0×10^{-4} (-1.1×10^{-3}–2.8×10^{-3})	.18
lr-width-R		1.00 (0.98–1.00)	1.00 (1.00–1.00)	1.00 (0.98–1.00)	.69
lr-neck-a		4.5×10^{-2} (3.3×10^{-2}–5.7×10^{-2})	8.1×10^{-2} (6.7×10^{-2}–9.4×10^{-2})	4.3×10^{-2} (3.3×10^{-2}–5.7×10^{-2})	.11
lr-neck-b		3.0×10^{-4} (-1.1×10^{-3}–2.4×10^{-3})	-1.9×10^{-3} (-2.0×10^{-3}–-1.7×10^{-3})	4.0×10^{-4} (-9.0×10^{-4}–-2.6×10^{-3})	.16
lr-neck-R		1.00 (0.99–1.00)	0.99 (0.99–0.99)	1.00 (0.98–1.00)	.64

Abbreviations: ACA, anterior cerebral artery; Acom, anterior communicating artery; AR, aspect ratio; BA, basilar artery; ICA, internal carotid artery; IQR, interquartile range; MCA, middle cerebral artery; mRS, modified Rankin Scale; Pcom, posterior communicating artery; SAH, subarachnoid hemorrhage; VA, vertebral artery.

[a] Clinical and morphological differences between ruptured and unruptured aneurysms were compared using Fisher's exact test for categorical variables and the Mann–Whitney U test for continuous variables.

[b] Race/ethnicity categories were investigator defined as Finnish, Japanese, or Other based on prior evidence from the PHASES score. No Finnish patients were included; therefore, the final categories analyzed were Japanese and Other. "Other" includes all patients not classified as Finnish or Japanese.

© 2025 Fujimura S et al. *JAMA Network Open.*

eTable 4. Patient and Aneurysm Characteristics in Institution D

Parameter	Patients	Aneurysms			
	Total	Total	Ruptured	Unruptured	P Value [a]
	Number (%) or Median (IQR)	Number (%) or Median (IQR)	Number (%) or Median (IQR)	Number (%) or Median (IQR)	
	N = 143	N = 201	N = 11	N = 190	
**Patient Characteristics**					
Age	59.1 (50.4–66.2)	59.2 (49.9–66.2)	62.3 (50.7–69.4)	58.8 (49.7–65.9)	.18
Sex (male)	31 (21.7%)	46 (22.9%)	3 (27.3%)	43 (22.6%)	.73
Multianeurysms	50 (35.0%)	101 (50.2%)	2 (18.2%)	99 (52.1%)	.03
Number of aneurysms	1 (1–2)	2 (1–3)	1 (1–1)	2 (1–3)	.02
Hypertension	74 (51.7%)	101 (50.2%)	6 (54.5%)	95 (50.0%)	.77
Alcohol consumption per week	74 (51.7%)	108 (64.3%)	8 (88.9%)	100 (62.9%)	.12
Smoking	55 (38.5%)	79 (39.3%)	6 (54.5%)	73 (38.4%)	.29
Number of cigarettes per day	10 (0–37.5)	10 (0–20)	10 (0–20)	10 (0–20)	.94
Years of smoking	13 (0–20)	14 (0–39)	24 (0–39)	13.5 (0–39)	.87
Hyperlipidemia	28 (19.6%)	33 (16.4%)	0 (0%)	33 (17.4%)	.13
Diabetes mellitus	8 (5.6%)	9 (4.5%)	1 (9.1%)	8 (4.2%)	.45
SAH	18 (12.6%)	26 (12.9%)	2 (18.2%)	24 (12.6%)	.60
Polycystic kidney disease	3 (2.1%)	5 (2.5%)	0 (0%)	5 (2.6%)	.59
Cerebral hemorrhage	0 (0%)	0 (0%)	0 (0%)	0 (0%)	1.00
Cerebral infarction	21 (14.7%)	30 (14.9%)	1 (9.1%)	29 (15.3%)	.58
Family history of SAH	29 (20.3%)	41 (20.4%)	0 (0%)	41 (21.6%)	.09
Family history of an aneurysm	21 (14.7%)	28 (13.9%)	2 (18.2%)	26 (13.7%)	.68
Family history of a polycystic kidney	2 (1.4%)	2 (1.0%)	0 (0%)	2 (1.1%)	.75
mRS (first visit)	1 (0–1)	1 (0–1)	0 (0–0.75)	1 (0–1.75)	.04
Race/ethnicity (Japanese)[b]	0 (0%)	0 (0%)	0 (0%)	0 (0%)	1.00

© 2025 Fujimura S et al. *JAMA Network Open.*

eTable 4. Patient and Aneurysm Characteristics in Institution D (continued)

**Aneurysm Characteristics**					
**Location**					
ICA (all sites)		47 (23.4%)	1 (9.1%)	46 (24.2%)	.25
ICA-Pcom		11 (5.5%)	0 (0%)	11 (5.8%)	.42
MCA (all sites)		90 (44.8%)	1 (9.1%)	89 (46.8%)	.02
ACA (all sites)		37 (18.4%)	6 (54.5%)	31 (16.3%)	.002
ACA-Acom		19 (9.5%)	5 (45.5%)	14 (7.4%)	<.001
BA (all sites)		20 (10.0%)	3 (27.3%)	17 (8.9%)	.05
BA-tip		0 (0%)	0 (0%)	0 (0%)	1.00
VA		7 (3.5%)	0 (0%)	7 (3.7%)	.52
**Morphological Parameters**					
Presence of irregularity		34 (17.4%)	6 (54.5%)	28 (15.2%)	.001
Length		3.5 (2.4–5.4)	9.0 (7.9–12.6)	3.4 (2.4–5.2)	<.001
Width		3.2 (2.1–4.7)	11.6 (7.9–12.9)	3.1 (2.1–4.4)	<.001
Neck diameter		2.3 (1.7–3.2)	3.7 (2.6–4.8)	2.2 (1.6–3.1)	.004
AR		1.14 (0.93–1.40)	1.10 (0.85–1.20)	1.14 (0.97–1.40)	.20
Saccularity		4.7 (2.7–9.4)	25.6 (18.5–36.8)	4.5 (2.6–8.6)	<.001

© 2025 Fujimura S et al. *JAMA Network Open*.

eTable 4. Patient and Aneurysm Characteristics in Institution D (continued)

**Aneurysm Characteristics**					
**Time-Dependent Morphological Parameters**					
Rate-length		1.9×10^{-4}<br>(-1.0×10^{-4}–8.0×10^{-4})	4.4×10^{-3}<br>(2.5×10^{-3}–4.5×10^{-3})	1.6×10^{-4}<br>(-1.2×10^{-4}–7.7×10^{-4})	.03
Rate-width		4.6×10^{-5}<br>(-1.6×10^{-4}–4.2×10^{-4})	2.0×10^{-3}<br>(7.5×10^{-4}–3.8×10^{-3})	0<br>(-2.5×10^{-4}–2.3×10^{-4})	.009
Rate-neck		2.5×10^{-5}<br>(-3.6×10^{-4}–3.2×10^{-4})	2.0×10^{-4}<br>(1.7×10^{-6}–2.7×10^{-4})	0<br>(-3.6×10^{-4}–3.2×10^{-4})	.66
lr-length-a		5.6×10^{-2}<br>(4.4×10^{-2}–8.1×10^{-2})	1.5×10^{-1}<br>(1.1×10^{-1}–1.9×10^{-1})	5.5×10^{-2}<br>(4.2×10^{-2}–7.8×10^{-2})	.03
lr-length-b		-6.5×10^{-4}<br>(-6.0×10^{-3}–5.3×10^{-3})	-1.5×10^{-2}<br>(-1.9×10^{-2}–-1.0×10^{-2})	5.0×10^{-5}<br>(-3.7×10^{-3}–6.0×10^{-3})	.01
lr-length-R		0.99<br>(0.96–1.00)	0.98<br>(0.96–0.99)	0.99<br>(0.96–1.00)	.56
lr-width-a		4.9×10^{-2}<br>(3.6×10^{-2}–7.8×10^{-2})	1.5×10^{-1}<br>(1.1×10^{-1}–1.6×10^{-1})	4.9×10^{-2}<br>(3.6×10^{-2}–7.3×10^{-2})	.06
lr-width-b		2.2×10^{-3}<br>(-1.4×10^{-3}–8.7×10^{-3})	-1.3×10^{-2}<br>(-1.4×10^{-2}–-1.2×10^{-2})	2.5×10^{-3}<br>(-1.8×10^{-4}–1.0×10^{-2})	.004
lr-width-R		0.98<br>(0.97–1.00)	0.97<br>(0.92–0.98)	0.98<br>(0.97–1.00)	.20
lr-neck-a		4.2×10^{-2}<br>(3.5×10^{-2}–5.3×10^{-2})	6.1×10^{-2}<br>(4.8×10^{-2}–7.2×10^{-2})	4.1×10^{-2}<br>(3.5×10^{-2}–5.0×10^{-2})	.24
lr-neck-b		2.3×10^{-3}<br>(-2.5×10^{-3}–7.8×10^{-3})	3.5×10^{-4}<br>(-2.0×10^{-3}–1.7×10^{-3})	3.2×10^{-3}<br>(-2.5×10^{-3}–9.2×10^{-3})	.43
lr-neck-R		0.98<br>(0.95–0.99)	0.98<br>(0.96–0.99)	0.98<br>(0.95–0.99)	.66

Abbreviations: ACA, anterior cerebral artery; Acom, anterior communicating artery; AR, aspect ratio; BA, basilar artery; ICA, internal carotid artery; IQR, interquartile range; MCA, middle cerebral artery; mRS, modified Rankin Scale; Pcom, posterior communicating artery; SAH, subarachnoid hemorrhage; VA, vertebral artery.

[a] Clinical and morphological differences between ruptured and unruptured aneurysms were compared using Fisher's exact test for categorical variables and the Mann–Whitney U test for continuous variables.

[b] Race/ethnicity categories were investigator defined as Finnish, Japanese, or Other based on prior evidence from the PHASES score. No Finnish patients were included; therefore, the final categories analyzed were Japanese and Other. "Other" includes all patients not classified as Finnish or Japanese.

© 2025 Fujimura S et al. *JAMA Network Open.*

eTable 5. Difference Between True Positive and False Negative

Parameter		Ruptured Median (IQR) True Positive N = 43 (All), 29 (B), 3 (C), and 11 (D)	Ruptured Median (IQR) False Negative N = 5 (All), 4 (B), 1(C), and 0 (D)	P Value[a]	Unruptured Median (IQR) True Positive N = 1010 (All), 741 (B), 186 (C), and 83 (D)	Unruptured Median (IQR) False Negative N = 442 (All), 261 (B), 75 (C), and 107(D)	P Value[a]
**Morphological Parameter**							
Length	Institution-B	6.0 (4.4–8.1)	1.9 (1.7–2.2)	0.004	2.0 (1.4–2.6)	3.9 (3.1–4.9)	<.001
	-C	6.7 (5.2–7.9)	3.3 (3.3–3.3)	0.44	2.1 (1.6–2.7)	5.1 (3.9–6.3)	<.001
	-D	9.0 (7.9–12.6)	-	-	2.3 (1.7–2.8)	4.8 (3.7–6.1)	<.001
Width	Institution-B	6.0 (4.8–8.1)	2.2 (2.0–3.0)	0.01	2.7 (2.2–3.5)	5.2 (4.4–6.5)	<.001
	-C	8.4 (6.4–9.0)	2.5 (2.5–2.5)	0.44	2.3 (1.8–3.0)	4.4 (3.7–6.1)	<.001
	-D	11.6 (7.9–12.9)	-	-	2.0 (1.5–2.7)	4.3 (3.2–5.5)	<.001
Neck	Institution-B	4.2 (3.4–5.5)	2.6 (2.3–3.0)	0.03	2.7 (2.2–3.3)	4.0 (3.2–5.0)	<.001
	-C	4.6 (3.6–5.9)	3.1 (3.1–3.1)	1.00	2.2 (1.9–3.0)	3.2 (2.5–3.8	<.001
	-D	3.7 (2.6–4.8)	-	-	1.7 (1.3–2.5)	2.7 (2.0–3.4)	<.001
AR	Institution-B	0.9 (0.8–1.2)	0.7 (0.6–1.0)	0.24	0.7 (0.6–0.9)	0.7 (0.6–0.9)	.09
	-C	0.8 (0.8–1.0)	1.3 (1.3–1.3)	0.44	0.9 (0.8–1.0)	1.1 (0.9–1.3)	<.001
	-D	1.1 (0.8–1.2)	-	-	1.1 (0.9–1.4)	1.1 (1.0–1.4)	.11
Saccularity	Institution-B	9.1 (5.7–12.1)	2.0 (1.1–3.0)	0.005	2.0 (1.3–2.8)	4.8 (3.7–6.8)	<.001
	-C	9.1 (7.6–12.8)	2.7 (2.7–2.7)	0.44	2.1 (1.4–3.0)	6.5 (4.7–9.4)	<.001
	-D	25.6 (18.5–36.8)	-	-	2.5 (2.0–3.2)	7.4 (5.3–13.0)	<.001
**Risk Score**							
PHASES score	Institution-B	9 (8–12)	8 (7–8)	0.03	5 (4–7)	5 (3–8)	.72
	-C	5 (3.5–5)	0 (0–0)	0.42	3 (1–5)	3 (1–5)	.14
	-D	7 (6–10)	-	-	3 (2–4.5)	3 (2–5)	.13
UCAS score	Institution-B	6 (5–7)	5 (4.5–5)	0.11	3 (1–4)	3 (1–4)	.03
	-C	3 (3–3.5)	0 (0–0)	0.42	3 (2–4)	3 (2–5)	.06
	-D	6 (4.5–8)	-	-	3 (2–4)	3 (3–4)	.09

[a] Clinical and morphological differences between ruptured and unruptured aneurysms were compared using Fisher's exact test for categorical variables and the Mann–Whitney U test for continuous variables.

© 2025 Fujimura S et al. *JAMA Network Open.*

eTable 6. Difference of the Parameters Between Institutions A, B, C, and D

**Number (%) or Median (Range)**							
**N = 4813 (All), 3312 (A), 1035 (B), 265 (C), and 201 (D)**							
**Morphological Parameter**				Saccularity	Institution-A	3.4 (2.5–4.8)	P value[a]
Length	Institution-A	2.8 (2.0–3.7)	P value[a]		-B	2.6 (1.6–4.0)	<.001
	-B	2.4 (1.6–3.4)	<.001		-C	2.7 (1.6–4.7)	<.001
	-C	2.7 (1.9–3.8)	.07		-D	4.7 (2.7–9.4)	<.001
	-D	3.5 (2.4–5.4)	<.001	**Morphological Parameter (Time-Dependent)**			
Width	Institution-A	3.4 (2.7–4.4)	P value[a]	Rate-length	Institution-A	2.1×10^{-5}(-1.1×10^{-4}–2.2×10^{-4})	P value[a]
	-B	3.2 (2.4–4.5)	<.001		-B	4.9×10^{-5} (-8.9×10^{-5}–3.1×10^{-4})	.003
	-C	2.8 (2.0–3.8)	<.001		-C	0 (-3.6×10^{-4}–5.5×10^{-4})	.70
	-D	3.2 (2.1–4.7)	<.001		-D	1.9×10^{-4} (-1.0×10^{-4}–8.0×10^{-4})	.009
Neck	Institution-A	3.3 (2.7–4.0)	P value[a]	Rate-width	Institution-A	4.8×10^{-5} (-1.7×10^{-4}–3.1×10^{-4})	P value[a]
	-B	3.0 (2.4–3.8)	<.001		-B	2.6×10^{-5} (-1.4×10^{-4}–3.3×10^{-4})	.49
	-C	2.5 (2.0–3.3)	<.001		-C	0 (-2.3×10^{-4}–5.6×10^{-4})	.54
	-D	2.3 (1.7–3.2)	<.001		-D	4.6×10^{-5} (-1.6×10^{-4}–4.2×10^{-4})	.98
AR	Institution-A	0.97 (0.81–1.15)	P value[a]	Rate-neck	Institution-A	2.7×10^{-5} (-1.5×10^{-4}–2.3×10^{-4})	P value[a]
	-B	0.73 (0.57–0.89)	<.001		-B	0 (-1.8×10^{-4}–2.2×10^{-4})	.14
	-C	0.93 (0.82–1.09)	.23		-C	0 (-3.0×10^{-4}–5.9×10^{-4})	.90
	-D	1.14(0.93–1.40)	<.001		-D	2.5×10^{-5} (-3.6×10^{-4}–3.2×10^{-4})	.92

[a] The Mann–Whitney U test was used to compare Institution-A with Institutions B, C, and D.

© 2025 Fujimura S et al. *JAMA Network Open.*

eTable 7. Comparison of Medical Imaging Between Institutions A, B, C, and D

**Medical Imaging**			**Total**		**Ruptured**		**Unruptured**	
			**Number (%) or Mean ± SD**		**Number (%) or Mean ± SD**		**Number (%) or Mean ± SD**	
			**N = 3,312 (A), 1,035 (B), 265 (C), and 201 (D)**		**N = 71 (A), 33 (B), 4 (C), and 11 (D)**		**N = 3,241 (A), 1,002 (B), 261 (C), and 190 (D)**	
Modality	CTA/DSA-CTA/DSA	Institution-A	2,014 (61.1%)	P value[a]	61 (85.9%)	P value	1,953 (60.5%)	P value[a]
		-B	494 (47.8%)	<.001	17 (51.5%)	<0.001	477 (47.7%)	<.001
		-C	196 (74.8%)	<.001	4 (100.0%)	0.44	192 (74.4%)	<.001
		-D	41 (20.5%)	<.001	6 (54.5%)	0.02	35 (18.5%)	<.001
	CTA/DSA-MRA	Institution-A	806 (24.4%)	P value[a]	0 (0.0%)	P value	806 (25.0%)	P value[a]
		-B	384 (37.1%)	<.001	2 (6.1%)	0.04	382 (38.2%)	<.001
		-C	9 (3.4%)	<.001	0 (0.0%)	1.00	9 (3.5%)	<.001
		-D	46 (23.0%)	.64	0 (0.0%)	1.00	46 (24.3%)	.84
	MRA-CTA/DSA	Institution-A	43 (1.3%)	P value[a]	4 (5.6%)	P value	39 (1.2%)	P value[a]
		-B	32 (3.1%)	<.001	7 (21.2%)	0.02	25 (2.5%)	.004
		-C	4 (1.5%)	.76	0 (0.0%)	0.65	4 (1.6%)	.63
		-D	9 (4.5%)	<.001	4 (36.4%)	0.002	5 (2.6%)	.09
	MRA-MRA	Institution-A	434 (13.2%)	P value[a]	6 (8.5%)	P value	428 (13.3%)	P value[a]
		-B	124 (12.0%)	.33	7 (21.2%)	0.07	117 (11.7%)	.20
		-C	53 (20.2%)	.001	0 (0.0%)	0.57	53 (20.5%)	.001
		-D	104 (52.0%)	<.001	1 (9.1%)	0.96	103 (54.5%)	<.001
Number of imaging sessions (all modalities)		Institution-A	6.2 ± 4.7	P value[a]	3.2 ± 3.4	P value	6.3 ± 4.7	P value[a]
		-B	3.8 ± 2.1	<.001	3.7 ± 2.3	0.05	3.8 ± 2.1	<.001
		-C	2.0 ± 0.7	<.001	3.0 ± 1.2	0.46	1.9 ± 0.6	<.001
		-D	2.2 ± 1.0	<.001	2.5 ± 1.4	0.82	2.2 ± 1.0	<.001
Imaging interval (all modalities) [month]		Institution-A	13.7 ± 10.3	P value[a]	7.7 ± 6.9	P value	13.8 ± 10.3	P value[a]
		-B	24.1 ± 20.0	<.001	13.9 ± 16.9	0.17	24.4 ± 20.0	<.001
		-C	38.3 ± 25.4	<.001	7.9 ± 7.0	0.90	38.9 ± 25.2	<.001
		-D	58.3 ± 37.9	<.001	13.3 ± 6.4	0.02	60.3 ± 37.5	<.001

[a] Fisher's exact test for categorical variables and Mann–Whitney U test for continuous variables were used to compare Institution-A with Institutions B, C, and D.

© 2025 Fujimura S et al. *JAMA Network Open.*

eFigure 1. Probability of Aneurysm Rupture

A

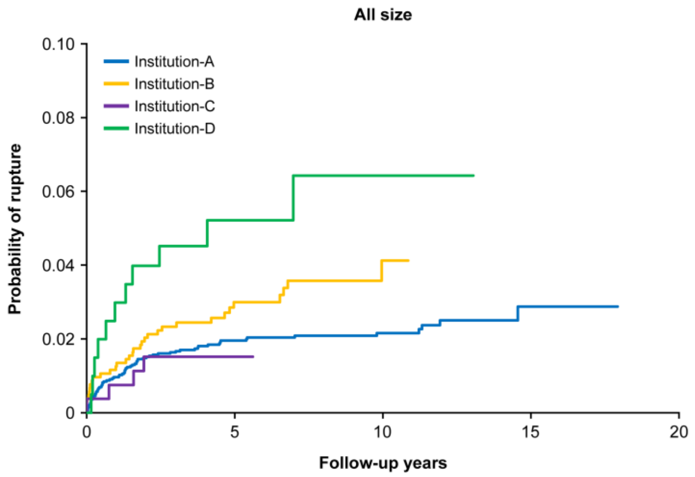

No. at risk

**Institution-A**	3312	2508	1295	193	0
**Institution-B**	1035	661	176	0	0
**Institution-C**	265	42	0	0	0
**Institution-D**	201	122	33	0	0

B

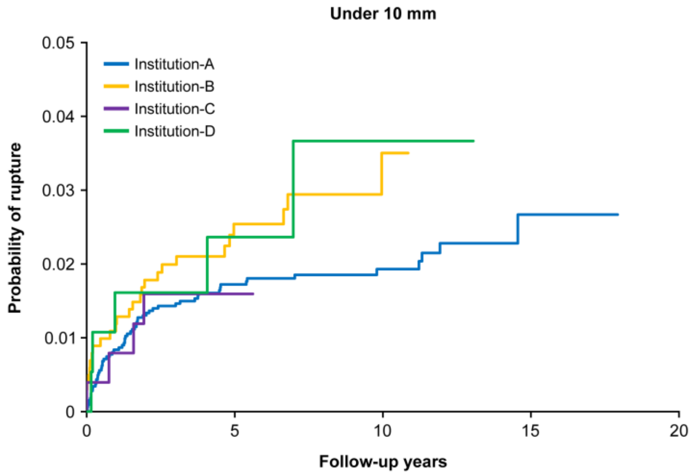

No. at risk

**Institution-A**	3223	2455	1261	185	0
**Institution-B**	1010	648	171	0	0
**Institution-C**	252	41	0	0	0
**Institution-D**	186	117	32	0	0

The probability of aneurysm rupture for all sizes (Panel A) and for aneurysms <10 mm (Panel B).

© 2025 Fujimura S et al. *JAMA Network Open*.

eFigure 2. Calibration Plot in the Development Cohort

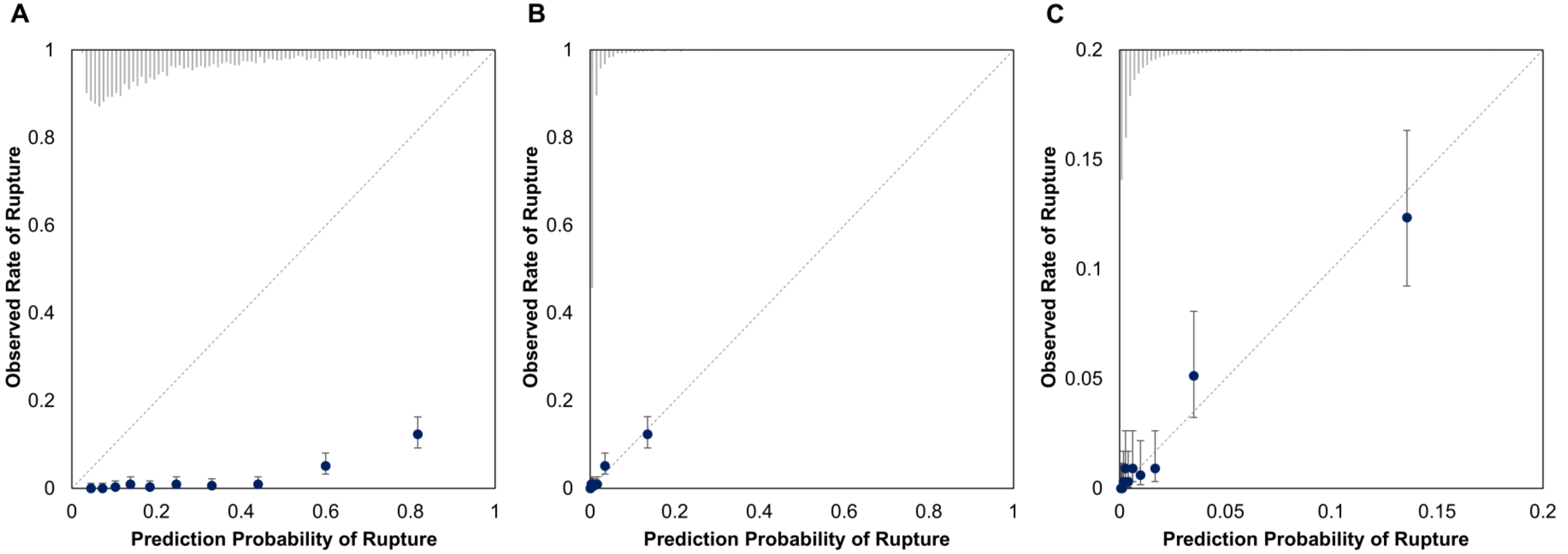

Each point represents the mean predicted probability and the observed rupture rate within a deciles bin; vertical bars indicate 95% CIs. The histogram shows the distribution of predicted probabilities. The uncalibrated calibration curve (Panel A), the logistic-recalibrated curve for the full probability range (0–1) (Panel B), and the logistic-recalibrated curve, zoomed view for the low-probability region (*x*=0–0.20; *y*=0–0.20) (Panel C) are shown.

© 2025 Fujimura S et al. *JAMA Network Open.*

eFigure 3. Feature Importance of the Developed MLM

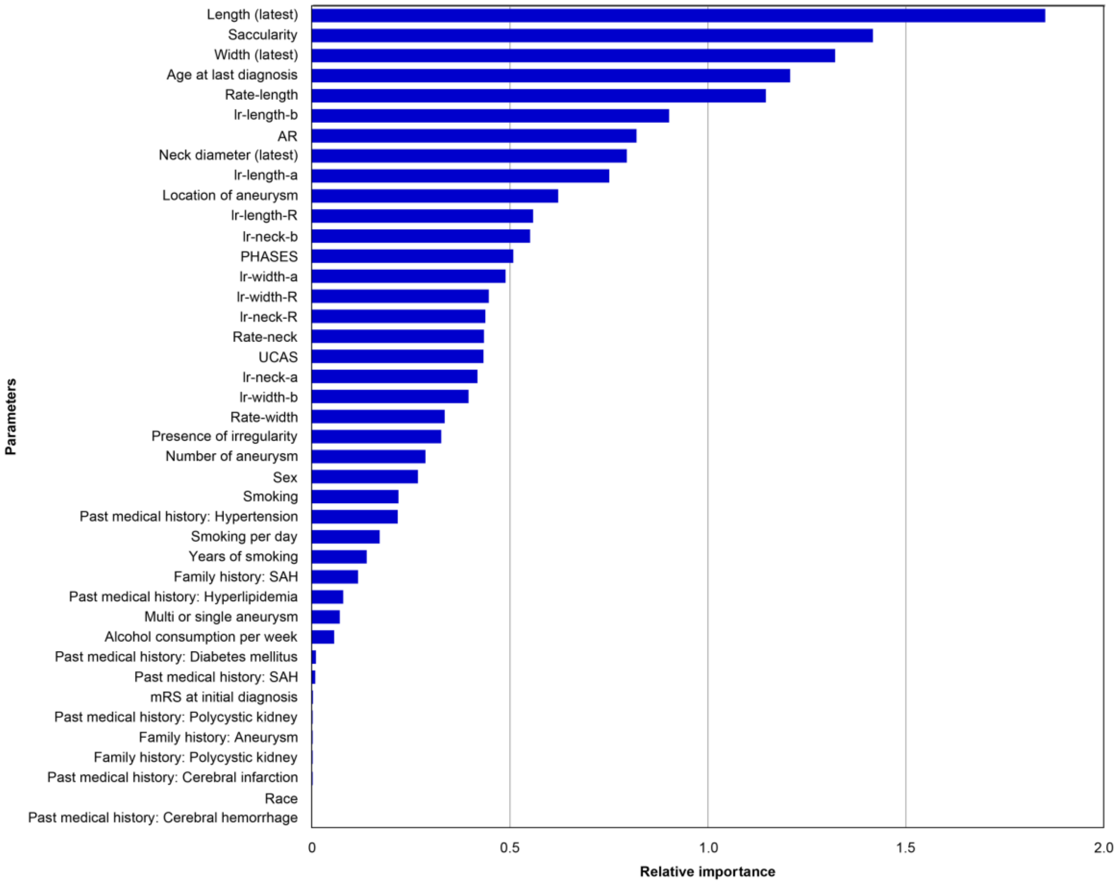

The relative importance indicates the contribution of each parameter to the predictions, but does not imply absolute importance or a specific tendency toward rupture risk.

© 2025 Fujimura S et al. *JAMA Network Open*.

eFigure 4. Calibration Plot in the External Validation Cohort

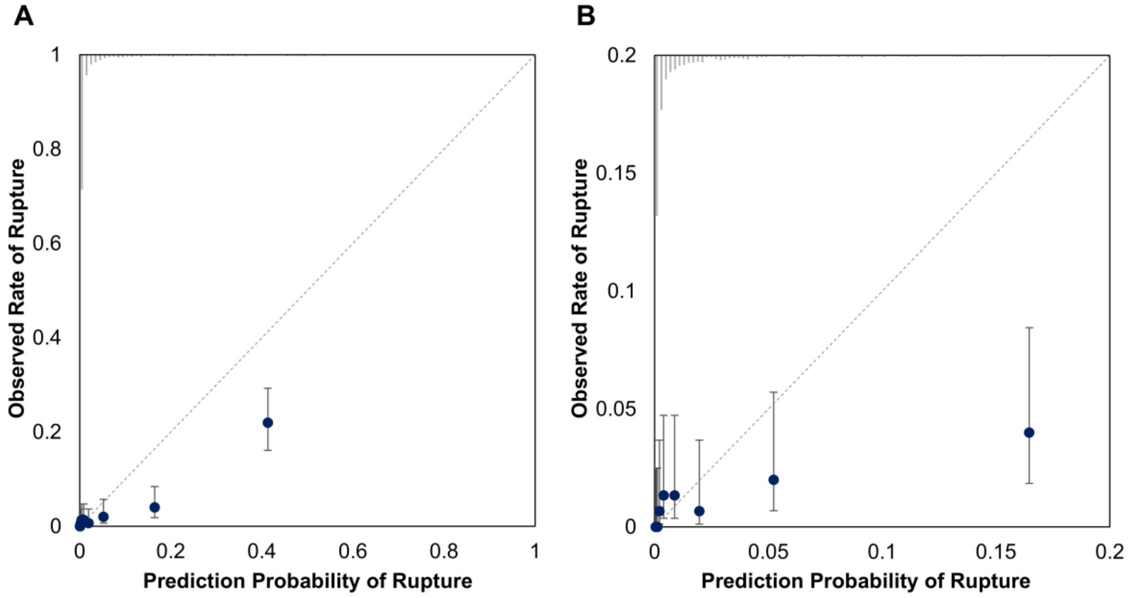

Each point represents the mean predicted probability and the observed rupture rate within a deciles bin; vertical bars indicate 95% CIs. The histogram shows the distribution of predicted probabilities. The logistic-recalibrated curve for the full probability range (0–1) (Panel A), and the logistic-recalibrated curve, zoomed view for the low-probability region (*x*=0–0.20; *y*=0–0.20) (Panel B) are shown.

© 2025 Fujimura S et al. *JAMA Network Open.*

eFigure 5. Case Summary of False-Negative Case 1 in Institution C

《 False negative case1 in institution-C 》

Age : 25 (at the Rupture prediction point)
Sex : Male
Aneurysm
Location : Lt. ICA-top
No. of aneurysm : 6
Presence of bleb : No
Alcohol consumption : Yes
Smoking : No
Past medical history : Sickle cell disease, SAH

History of imaging :

[Date]	[Length]	[Width]	[Neck]	[Modality]
19 months B.R.	3.3 mm	2.5 mm	3.1 mm	DSA
└ Rupture prediction point				
Date of rupture	5.5 mm	5.7 mm	3.7 mm	DSA

*B.R.: Before Rupture

mRS (First visit) : 3
mRS (After SAH) : 1

Image at 19 months B.R.

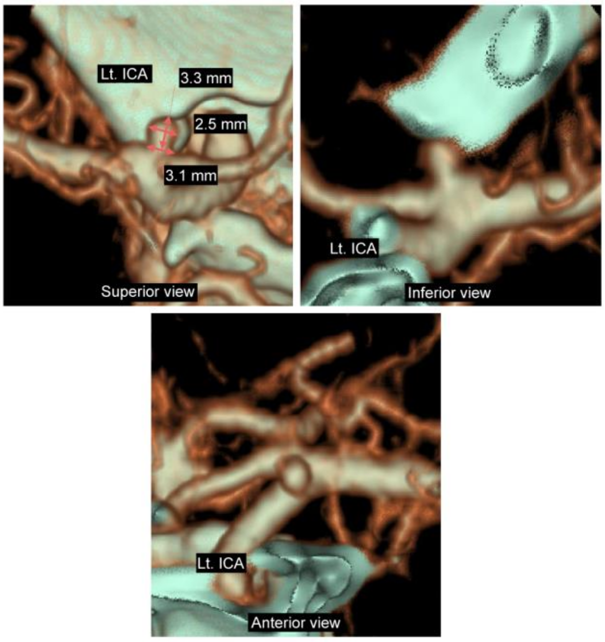

eFigure 6. Case Summary of False-Negative Case 2 in Institution B

《 False negative case2 in institution-B 》

Age : 72 (at the Rupture prediction point)
Sex : Female
Aneurysm
Location : Rt. MCA-bifurcation
No. of aneurysm : 1
Presence of bleb : No
Alcohol consumption : No
Smoking : Yes
Smoking per day : 20
Years of smoking : 50
Past medical history : Hypertension, Cerebral infarction, Chronic kidney disease

History of imaging :

[Date]	[Length]	[Width]	[Neck]	[Modality]
5 days B.R.	1.3 mm	2.3 mm	2.6 mm	MRA
└ Rupture prediction point				

*B.R.: Before Rupture

mRS (First visit) : 3
mRS (After SAH) : 6

Image at 5 days B.R.

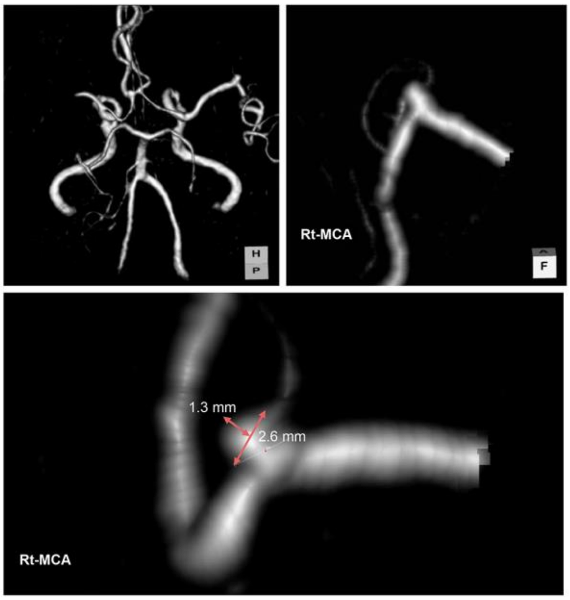

© 2025 Fujimura S et al. *JAMA Network Open*.

eFigure 7. Case Summary of False-Negative Case 3 in Institution B

《 False negative case3 in institution-B 》

Age : 59 (at the Rupture prediction point)
Sex : Female
Aneurysm
Location : Lt. ICA-Pcom
No. of aneurysm : 2
Presence of bleb : No
Alcohol consumption : No
Smoking : No
Past medical history : Hypertension, SAH, Arthrorheumatism

History of imaging :

[Date]	[Length]	[Width]	[Neck]	[Modality]
83 months B.R.	0.6 mm	0.6 mm	0.6 mm	3D-CTA
70 months B.R.	1.0 mm	1.0 mm	1.3 mm	3D-CTA
34 months B.R.	3.4 mm	3.0 mm	2.9 mm	3D-CTA
10 months B.R.	1.8 mm	1.6 mm	2.6 mm	MRA
└ Rupture prediction point				
Date of rupture	3.4 mm	3.0 mm	3.0 mm	3D-CTA

*B.R.: Before Rupture

mRS (First visit) : 3
mRS (After SAH) : 6

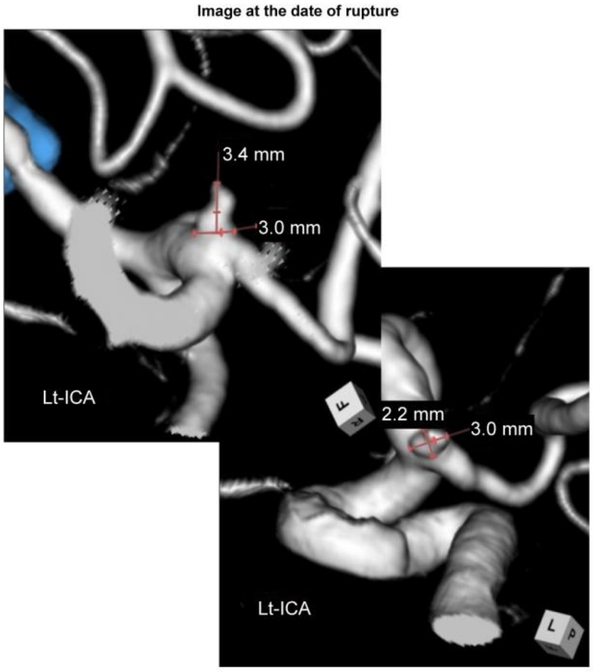

eFigure 8. Case Summary of False-Negative Case 4 in Institution B

《 False negative case4 in institution-B 》

Age : 48 (at the Rupture prediction point)
Sex : Female
Aneurysm
Location : Rt. ACA-distal
No. of aneurysm : 1
Presence of bleb : Yes
Alcohol consumption : No
Smoking : No
Past medical history : Polycystic kidney

History of imaging :

[Date]	[Length]	[Width]	[Neck]	[Modality]
32 months B.R.	2.0 mm	1.6 mm	1.6 mm	3D-CTA
30 months B.R.	1.9 mm	2.7 mm	1.7 mm	3D-CTA
24 months B.R.	1.9 mm	2.2 mm	1.4 mm	3D-CTA
12 months B.R.	1.9 mm	2.1 mm	1.4 mm	3D-CTA
└ Rupture prediction point				
Date of rupture	3.5 mm	3.1 mm	1.1 mm	3D-CTA

*B.R.: Before Rupture

mRS (First visit) : 0
mRS (After SAH) : 1

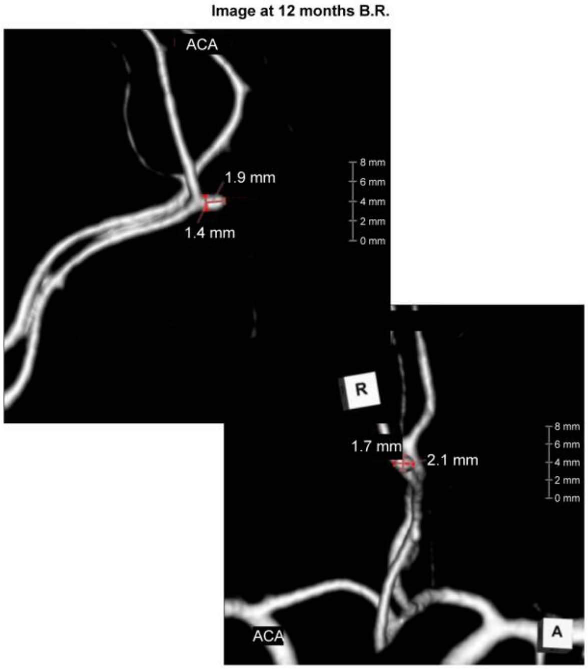

© 2025 Fujimura S et al. *JAMA Network Open*.

eFigure 9. Case Summary of False-Negative Case 5 in Institution B

《 False negative case5 in institution-B 》

Age : 73 (at the Rupture prediction point)
Sex : Female
Aneurysm
Location : ACA-Acom
No. of aneurysm : 1
Presence of bleb : No
Alcohol consumption : No
Smoking : No
Past medical history : Arthrorheumatism

History of imaging :

[Date]	[Length]	[Width]	[Neck]	[Modality]
119 months B.R.	3.2 mm	4.8 mm	4.0 mm	MRA
112 months B.R.	3.1 mm	4.0 mm	4.5 mm	3D-CTA
88 months B.R.	3.0 mm	4.8 mm	4.0 mm	3D-CTA
45 months B.R.	3.1 mm	4.9 mm	3.6 mm	3D-CTA
31 months B.R.	3.2 mm	4.8 mm	4.0 mm	MRA
21 months B.R.	3.1 mm	5.0 mm	4.0 mm	MRA
7 months B.R.	2.9 mm	5.0 mm	4.0 mm	MRA

└ Rupture prediction point

*B.R.: Before Rupture

mRS (First visit) : 0
mRS (After SAH) : 6

Image at 7 months B.R.

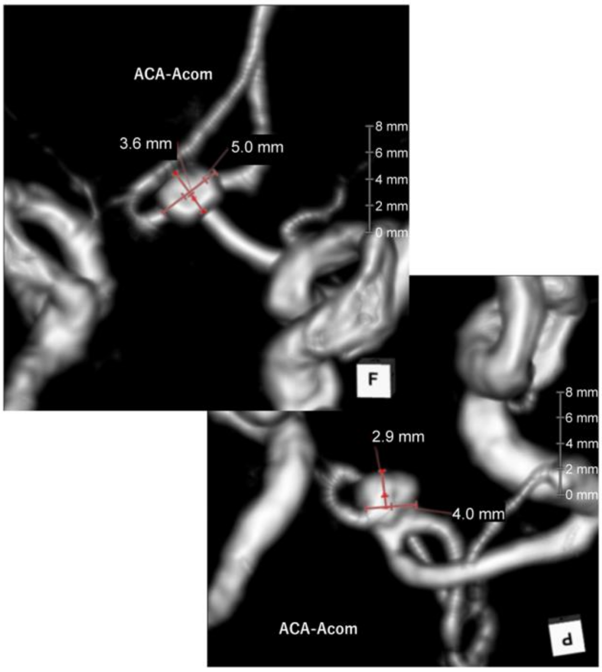

© 2025 Fujimura S et al. *JAMA Network Open*.
