# Supplementary material for: Development and Validation of a Prediction Model for Intracranial Aneurysm Rupture Risk
Source: JAMA Netw Open. 2025 Dec 23;8(12):e2550772. doi: 10.1001/jamanetworkopen.2025.50772 (PMC12728650; doi:10.1001/jamanetworkopen.2025.50772)
Supplement: Supplement 2. — Data Sharing Statement [file jamanetwopen-e2550772-s002.pdf]

Data Sharing Statement

Fujimura. Development and Validation of a Prediction Model for Intracranial Aneurysm Rupture Risk. *JAMA Netw Open*. Published December 23, 2025.
doi:10.1001/jamanetworkopen.2025.50772

Data
Data available: Yes
Data types: Deidentified participant data
How to access data: ymurayama@jikei.ac.jp
When available: With publication

Supporting Documents
Document types: None

Additional Information
Who can access the data: Researchers whose requested the data and proposed use of the data has been approved.
Types of analyses: For a specified purpose.
Mechanisms of data availability: With investigator support and after approval of a proposal.
Any additional restrictions: The data contain sensitive clinical information, and access is limited to non-commercial academic use under data-sharing agreements approved by the corresponding institution.
